# *In situ* mutation detection and visualization of intratumor heterogeneity for cancer research and diagnostics

**DOI:** 10.18632/oncotarget.1527

**Published:** 2013-11-21

**Authors:** Ida Grundberg, Sara Kiflemariam, Marco Mignardi, Juliana Imgenberg-Kreuz, Karolina Edlund, Patrick Micke, Magnus Sundström, Tobias Sjöblom, Johan Botling, Mats Nilsson

**Affiliations:** ^1^ Department of Immunology, Genetics and Pathology, Science for Life Laboratory, Rudbeck Laboratory; ^2^ Present address: Olink Bioscience, Uppsala Science Park, Uppsala, Sweden; ^3^ Science for Life Laboratory, Department of Biochemistry and Biophysics, Stockholm University; ^4^ Present address: Department of Medical Sciences, Uppsala University, Uppsala, Sweden; ^5^ Present address: Leibniz Research Centre for Working Environment and Human Factors (IfADo) at Dortmund TU, Dortmund, Germany

**Keywords:** Padlock probes, RCA, in situ, KRAS, cancer diagnostics

## Abstract

Current assays for somatic mutation analysis are based on extracts from tissue sections that often contain morphologically heterogeneous neoplastic regions with variable contents of genetically normal stromal and inflammatory cells, obscuring the results of the assays. We have developed an RNA-based *in situ* mutation assay that targets oncogenic mutations in a multiplex fashion that resolves the heterogeneity of the tissue sample. Activating oncogenic mutations are targets for a new generation of cancer drugs. For anti-EGFR therapy prediction, we demonstrate reliable *in situ* detection of *KRAS* mutations in codon 12 and 13 in colon and lung cancers in three different types of routinely processed tissue materials. High-throughput screening of *KRAS* mutation status was successfully performed on a tissue microarray. Moreover, we show how the patterns of expressed mutated and wild-type alleles can be studied *in situ* in tumors with complex combinations of mutated *EGFR*, *KRAS* and *TP53*. This *in situ* method holds great promise as a tool to investigate the role of somatic mutations during tumor progression and for prediction of response to targeted therapy.

## INTRODUCTION

Therapeutic targeting of oncogenic mutations in signal transduction pathways has opened a new era in oncology and created a need for efficient mutational analyses in routine pathology. Presently, the complexity of cancer tissues is not taken into account in clinical mutation diagnostics which is performed on crude tissue extracts. Therefore, all different cell types present in a tumor sample – normal parenchymal cells, stromal cells, inflammatory cells, pre-neoplastic and fully developed malignant cells – contribute their wild-type and mutated alleles to the analysis. Thanks to next generation sequencing technology, knowledge about the genetic heterogeneity within a single cancer lesion with regard to acquired aberrations is increasing rapidly. The data support the concept of clonal evolution where different mutations can be enriched in different sub-clones due to an array of selection mechanisms at work in different compartments of the tumor bulk – hypoxia, inflammation, necrosis, inflammation and organ specific environmental factors [1]. To characterize intratumor heterogeneity in a routine diagnostic setting, tumor cells can be enriched by manual microdissection, but more detailed analysis of specific tumor regions requires laborious laser-assisted microdissection. Hence, methods that offer *in situ* mutation detection directly on tissue sections are highly warranted.

Recently, we published a novel strategy for *in situ* detection and genotyping of individual mRNA molecules [2]. In this approach, target transcripts are first converted into cDNA molecules and thereafter detected using padlock probes and target primed rolling-circle amplification (RCA). Padlock probes are short linear oligonucleotides that become circular when the ends are brought together by hybridization to a target sequence, and joined by a DNA ligase if perfectly matched [3-6]. The padlock probes contain tag sequences that after amplification act as detection sites for fluorescently labeled oligonucleotides. The resulting rolling circle products (RCPs) appear as bright signals localized in the cytoplasm of the cells. Thus, this *in situ* technique offers single transcript analysis directly on slides and circumvents traditional DNA extraction from heterogeneous tumor tissues. In addition to point mutations and single-nucleotide polymorphisms (SNPs), the method can provide information on RNA-edited transcripts, tissue specific allele expression [2], alternative splicing, fused transcript variants and small insertions/deletions [7].

The aim of this study was to develop an *in situ* assay for mutation analysis in clinical oncology and diagnostic molecular pathology, especially with regard to use in routinely collected formalin–fixed, paraffin-embedded (FFPE) tissue. A primary goal of the present study was to design a multiplexed *in situ* mutation detection assay for point mutations in *KRAS,* one of the most frequently activated oncogenes in cancer. In colorectal cancer, the presence of mutations in the *KRAS* gene indicates that the tumor will not respond to EGFR antibody therapy [8]. There are seven point mutations in codon 12 and 13 that together account for approximately 95% of all *KRAS* mutations in colorectal cancer [9]. In lung adenocarcinoma *KRAS* mutations are associated with poor prognosis and non-responsiveness to EGFR inhibitors whereas *KRAS* wild-type tumors with *EGFR* mutations are linked to better prognosis and response to EGFR inhibitors [10]. The secondary goal of this study was to apply the technique to explore if specific mutations are present in separate cancer sub-clones, and if differences in the balance between expressed mutated and wild-type alleles can be linked to any geographical areas or histologic patterns in a cancer lesion. To this end, we designed individualized patient-specific *in situ* assays for tumors with multiple known oncogene mutations selected from a cohort [11] of lung cancer cases with characterized mutations in *EGFR*, *KRAS* and *TP53* [12].

## RESULTS

### Assay design

**Figure 1:**
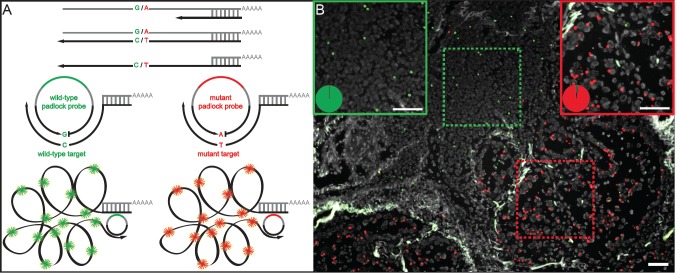
*In situ* genotyping with padlock probes and target-primed RCA (A) Schematic overview of the method. Target cDNA (black) is created by reverse transcription with an LNA-primer. Target mRNA (grey) is degraded by RNase H, except for the region that is hybridized to the LNA-part of the primer that is protected from degradation, anchoring the created cDNA to the target. Target specific padlock probes (wild-type:green, mutant:red), with similar target sites except for the single point mutated base (e.g. G/A), are hybridized to the cDNA and circularized by target-dependent ligation. The targeted transcripts act as primer for RCA and the resulting RCPs are labeled with fluorescence-labeled detection probes and visualized as bright spots in the cells or tissue. (B) *In situ* detection of a *KRAS* codon 12 (G12S) mutation in fresh frozen lung tumor tissue. Green RCPs represent wild-type *KRAS* transcripts (GGT), red RCPs represent mutant *KRAS* (AGT). The green dashed square indicates normal lymphocytes (magnified in solid green square) and the red dashed square indicates tumor cells with an activating G12S *KRAS* mutation (magnified in solid red square). The pie charts indicate the ratio between wild-type (green) and mutant (red) signals in respective square. Cell nuclei are shown in grey. Scale bar, 50 μm.

We designed padlock probes for point mutations in *KRAS* codons 12, 13 (G12S, G12R, G12C, G12D, G12A, G12V and G13D) and 61 (Q61H), as well as for *EGFR* (G719A, G719C, S768I and L858R) and *TP53* (S127F and P190S). Padlock probes for the wild-type forms of the different targets were designed as well (Supplementary Table 1 and 2). The mutation-specific padlock probes were designed with identical target sequences except for the last nucleotide in the 3´-end that differ depending on genotype (Fig. 1A). Mismatches at this position are not accepted by the DNA ligase used and single nucleotide differences, like point mutations, are therefore efficiently discriminated [13]. To distinguish the RCPs from each other using detection probes labeled with different fluorescence dyes, e.g. green and red, two different sites for detection probes for wild-type and mutant padlocks were incorporated. We also included detection of the *ACTB* transcript in our assays, detected by an additional fluorophore, as an internal reference having a relative constant expression between cell types. A comparison of the *ACTB* signals across samples provided an estimation of the detection efficiency in different samples. The *ACTB* data was useful during the development phase of this assay, but turned out to be dispensable for mutation scoring and tissue classification. Before applying the padlock probes onto cell lines or tissues they were evaluated *in vitro* with synthetic templates to assure similar hybridization and ligation efficiency.

### Validation of *KRAS in situ* mutation detection in colon and lung cancer tissues with known *KRAS* status

**Figure 2:**
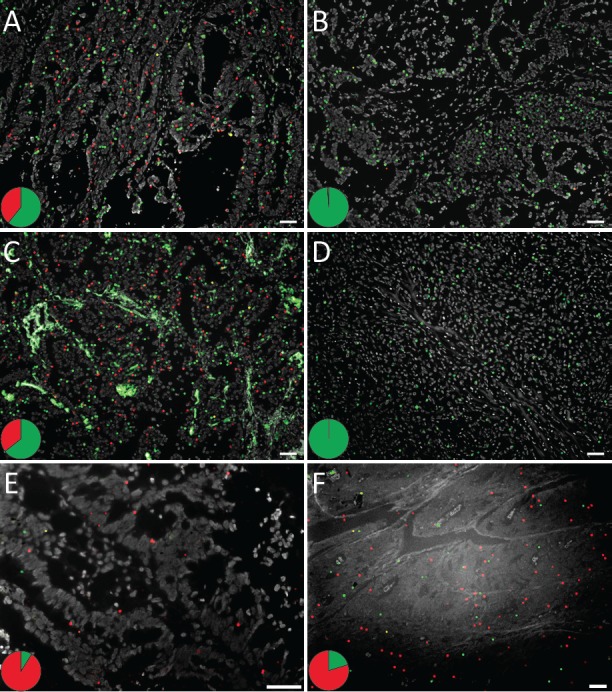
*In situ* mutation detection of codon 12 and 13 *KRAS* mutations on (A-D) fresh frozen colon and lung tissues and (E, F) FFPE colon tissues using padlock probes and RCA The tissues display *KRAS* mutant (red) and wild-type (green) RCPs. Cell nuclei are shown in grey. *KRAS* G12D mutation analysis in fresh frozen (A) mutant and (B) wild-type colon tumor tissue, in (C) mutant and (D) wild-type lung tumor tissue, and on FFPE colon tissues with reported (E) G12C or (F) G13D *KRAS* mutations. The pie charts indicate the ratio between wild-type (green) and mutant (red) signals in respective tissue. The images were acquired with 10x or 20x objective. Scale bar, 50 μm. See Supplementary Fig. 2-4 for the complete set of analyzed samples.

The selectivity of the padlock probes was first tested *in situ* on *KRAS* wild-type- and mutant cell lines (Supplementary Fig. 1). After confirmation of the quality of the probes, the *in situ* genotyping method was applied to ten fresh frozen human colon and lung cancer tissues with known *KRAS* status (Fig. 2A-D and Supplementary Fig. 2 and 3). In this validation phase, each mutation specific probe-pair was tested individually. The samples represented all codon 12 and 13 mutations except for the rarest one, G12R (Table 1), but the performance of the G12R mutation assay was verified on one of the tested cell lines (Supplementary Fig. 1F). Scoring for *KRAS* status was done by microscopic inspection in a fashion similar to regular fluorescent *in situ* hybridization (FISH). Scoring criteria are further discussed in Supplementary Note 2.

**Table 1:** Summary of mutation analysis performed on fresh frozen and FFPE samples

Sample ID	Sample Type	Target	1. Pyros equencing		2. *In situ* mutation detection	
			Mutants/Total	Mutations	Mutants/Total	Concordance
**1-5**	Fresh frozen colon	*KRAS*	4/5	1xG12D, 1xG12C, 1xG13D, 1xG12A	4/5	100%
**6-10**	Fresh frozen lung	*KRAS*	4/5	1xG12D, 1xG12V, 1xG12C, 1xG12S	4/5	100%
**11-24**	FFPE colon	*KRAS*	14/14	2xG12D, 3xG12V, 2xG12C, 3xG13D, 2xG12S, 1xG12A	14/14	100%
**25-26**	FFPE lung	*FRAS*	2/2	2xQ61H	2/2	100%
**27-35**	FFPE lung	*EGFR*	8/9	8xL858R	8/9	100%
**36-40**	FFPE core needle biopsies, lung	*EGFR*	2/5	L858R	2/5	100%
**41**	FFPE lung	*EGFR*	1/1	1xG719C, 1xS768I	1/1	100%
**42**	FFPE lung	*EGFR/TP53*	1/1	1xG719A, 1xS127F	1/1	100%
**43**	FFPE lung	*KRAS/TP53*	1/1	1xG12C, 1xP190S	1/1	100%

The colon and lung sections with *KRAS* mutations displayed both wild-type and mutant signals (Fig. 2A, C and Supplementary Fig. 2A-D and 3A-D), whereas wild-type tissues almost exclusively showed wild-type signals (Fig. 2B, D and Supplementary Fig. 2E and 3E). By visually examining the ten samples we could clearly see variations in *KRAS* expression levels both within and between the tissues. Most cases displayed both wild-type and mutant *KRAS* signals in the tumor cell areas, indicating the presence of a heterozygous mutation and expression of both alleles. In contrast, one lung sample predominantly displayed mutant signals in the tumor regions, potentially reflecting *KRAS* homozygous mutation or loss-of-heterozygosity (LOH), while the few existing wild-type signals originated from the stroma. (Fig. 1B and Supplementary Fig. 3D).

We next assessed whether the *in situ* padlock probe technique could be applied on FFPE tissues. *KRAS* mutation analysis was performed on a collection of 14 colorectal FFPE cancer tissues (Fig. 2E, F, Table 1 and Supplementary Fig. 4) with known *KRAS* mutations in codon 12 and 13. We also designed probes for the most common mutation in codon 61 (Q61H) and tested them in two FFPE colon tumor samples (Table 1 and Supplementary Fig. 5). All tissues displayed a combination of wild-type and mutant signals, however variation in the number of signals (for both *KRAS* and *ACTB*) was significant between tissues, which probably reflects the expected difference in tissue quality among FFPE samples.

### Multiplex *in situ* detection of *KRAS* mutations on prospective clinical samples with unknown mutation status

**Figure 3:**
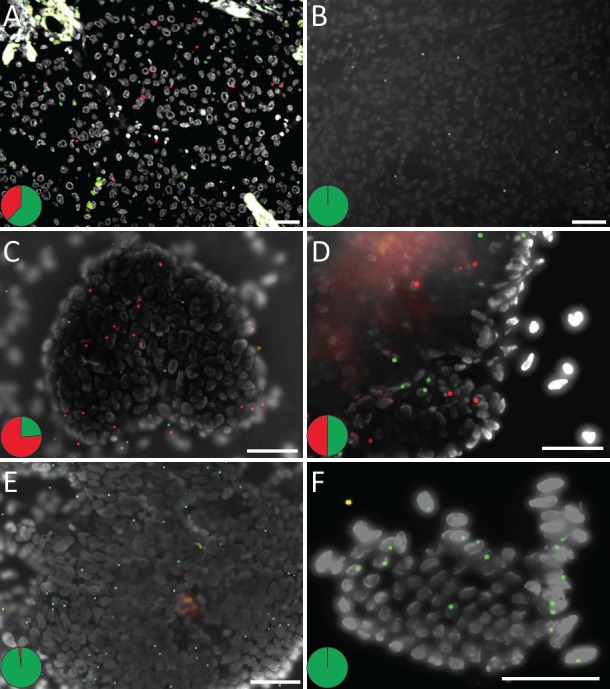
*In situ* detection of *KRAS* mutations on prospective clinical samples with unknown mutation status The tissues display *KRAS* mutant (red) and wild-type (green) RCPs and cell nuclei are shown in grey. *KRAS* detection in lung FFPE tissues with (A) a G12C mutation and in (B) a *KRAS* wild-type sample. Mutation detection in prospective colon touch tumor imprints in (C) G12D and (D) G12R mutated samples and in (E, F) imprint samples with wild-type *KRAS*. The pie charts indicate the ratio between wild-type (green) and mutant (red) signals in respective tissue. Scale bar, 50 μm. See Supplementary Fig. 7 and 8 for the complete set of analyzed samples.

To investigate whether multiple mutations could be tested in the same reaction, we combined all *KRAS* probes and compared the *in situ* mutation detection result to that of individual mutation-specific probes (Supplementary Fig. 6). Detection efficiency or selectivity was not affected noticeably when multiple probes competed for the two-codon target site. The combined analysis can thus provide a rapid answer to whether the tumor harbors an activating *KRAS* mutation or not. Multiplex mutation detection was thereafter demonstrated on eight prospective lung FFPE tissues (Table 2 and Supplementary Fig. 7) with unknown *KRAS* mutation status. *In situ* mutation analysis suggested that three of the eight cases were mutated (Fig. 3A and Supplementary Fig. 7A-C). The results were compared to pyrosequencing of DNA extracts from the same tissues, and the suggested genotypes were confirmed to be correct in every case (Supplementary Fig. 12).

**Table 2:** Summary of mutation analysis performed on prospective FFPE and tumor imprint samples

Sample ID	Sample Type	Target	1. *In situ* mutation detection	2. Pyrosequencing		
			Mutants/Total	Mutants/Total	Mutations	Concordance
**44-51**	FFPE lung	*KRAS*	3/8	3/8	2xG12C, 1xG12R	100%
**52-59**	Colon tumor imprint	*KRAS*	2/8	2/8	1xG12D, 1xG12R	100%
**60-84**	FFPE colon (from TMA)	*KRAS*	11/25	11/25	6xG12V, 2xG12S, 2xG13D, 1xG12A	100%

To test the method in a diagnostic setting involving cytology preparations, we prepared tumor imprint slides from eight prospective fresh colon cancer specimens with unknown *KRAS* mutation status, and subjected them to multiplex *KRAS in situ* mutation detection (Fig. 3C-F, Table 2 and Supplementary Fig. 8). Two cases were scored positive in the *in situ* mutation assay (Fig. 3C, D and Supplementary Fig. 8A, B), while the other six only showed wild-type signals (Fig. 3E, F and Supplementary Fig. 8C-H). DNA from corresponding FFPE tumor sections were thereafter tested for *KRAS* mutations by pyrosequencing, and the results were concordant with the *in situ* assay (Supplementary Fig. 12).

### High-throughput mutation screening on tissue microarrays

Tissue microarrays (TMA) can be used to analyze hundreds of FFPE tumor samples on one slide, and have been used to characterize protein expression by immunohistochemistry (IHC) and gene copy number variations (by FISH) in large patient cohorts [14]. We assayed a TMA containing 25 duplicate FFPE colon samples (normal mucosa, tubular adenomas, serrated adenomas, primary tumors, and matched metastases) of unknown *KRAS* mutation status for *KRAS* codon 12 and 13 mutations. Eleven samples, two adenomas, one serrated adenoma, four primary tumors and their matched metastases, were scored *KRAS* positive (Supplementary Fig. 9). Subsequent mutational analysis of *KRAS* by pyrosequencing on the corresponding FFPE blocks was concordant with the *in situ* data (Supplementary Fig. 12).

### Differential expression of mutated oncogene alleles related to tumor progression and histological heterogeneity

**Figure 4:**
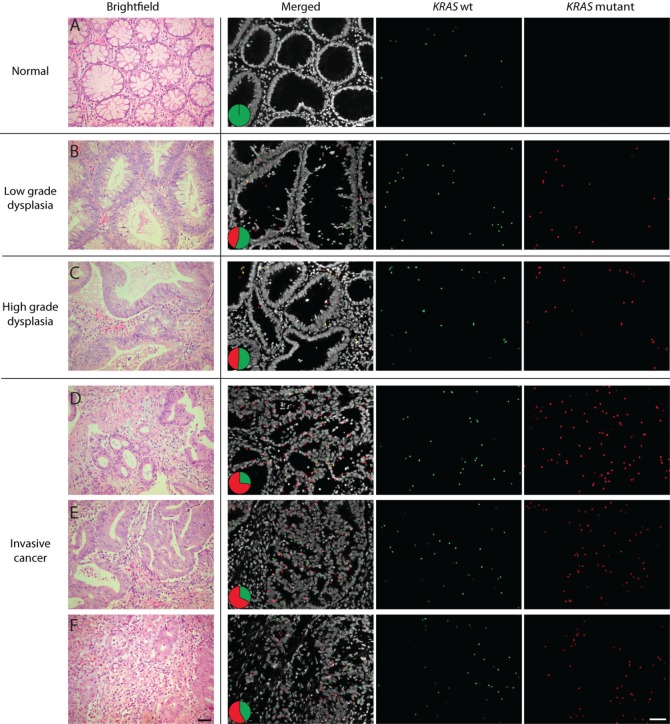
Detection of the Q61H *KRAS* point mutation in a FFPE colon sample Images show representative regions of (A) normal colon, (B) low grade dysplasia, (C) high grade dysplasia and (D-F) invasive cancer. The figure presents brightfield microscopy images from corresponding tumor areas in a consecutive H&E-stained slide to the left and fluorescent images to the right in a merged format as well as in respective color to show the distribution of the target transcripts. Red RCPs show mutant *KRAS* and wild-type RCPs are shown as green spots. The pie charts indicate the ratio between wild-type (green) and mutant (red) signals in respective tissue area. Nuclei are shown in grey. Scale bars, 50 μm.

Variable expression of a mutated oncogene across a tumor could potentially result in a variable response to targeted therapy in different areas of a tumor. We therefore screened cases analyzed by the *in situ* assay for distinct patterns of expressed mutations. In one colon cancer case with a codon 61 mutation, the histological progression from normal colon mucosa to low-grade and high-grade dysplasia and invasive carcinoma could be visualized on a single slide (Fig. 4 and Supplementary Fig. 5A). There was a clear increase in the expression of mutated mRNA transcripts, relative to the wild-type transcript and in absolute numbers in a given tumor cell area, along with transition from adenomatous to invasive tumor growth. Thus, one can speculate if the level of resistance to EGFR inhibitors would follow the expression levels of mutant *KRAS* transcripts in the different tumor compartments.

**Figure 5:**
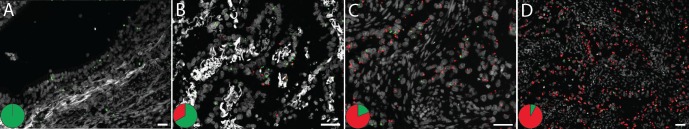
Detection of the *EGFR* L858R mutation in a FFPE lung tumor tissue sample using mutant and wild-type specific L858R padlock probes and RCA Red RCPs represent mutants and green RCPs wild-type *EGFR.* Cell nuclei are shown in grey. The figure illustrates (A) a small bronchus with respiratory epithelium with expression of wild-type *EGFR* transcripts and (B-D) great heterogeneity within a tumor with regard to expression of the mutant *EGFR* L858R transcript. The pie charts indicate the ratio between wild-type (green) and mutant (red) signals in respective tissue area. Scale bar, 50 μm.

We also targeted the *EGFR* L858R mutation in a set of nine surgical FFPE lung cancer specimens in which eight were known to be positive (Table 1). The results from the *in situ* mutation assay were concordant with the DNA sequencing data. Even though some of the lung samples were aged more than a decade, we observed high detection efficiency with high numbers of signals, especially mutant signals, which might reflect high mRNA expression from amplified *EGFR* in the tumor (Supplementary Fig. 10). In one lung sample we observed a great histological heterogeneity with regard to tumor growth patterns (Fig. 5 and Supplementary Fig. 10A). In normal bronchial epithelium only wild-type *EGFR* signals were detected (Fig. 5A). In areas with bronchioalveolar/lepidic growth pattern (Fig. 5B) the expression of mutated *EGFR* was low, and equaled the expression of the wild-type allele. The expression of the mutant allele was increased in more poorly differentiated glandular areas, both in absolute numbers and relative to the wild-type allele (Fig. 5C) and peaked in areas with solid growth pattern (Fig. 5D). Thus, if the expression of L858R transcripts affects the sensitivity of a tumor clone for EGFR-TKI (tyrosine kinase inhibitor) therapy, as would be expected based on IHC data [15], the poorly differentiated areas would be expected to respond better than the well differentiated areas in this individual tumor. To evaluate if the method is applicable to small diagnostic samples, we analyzed five core needle biopsies from lung cancers, of which two were known to be positive for the L858R mutation (Supplementary Fig. 10J-N). Despite low tumor cell content, as low as 10% in one of the cases, all five biopsies were correctly scored using the *in situ* assay (Table 1). The specificity and sensitivity of the assay was further evaluated in experiments in which cells from a *KRAS* mutant (G12S) cell line (A-549) were mixed in different ratios with a *KRAS* wild-type cell line (ONCO-DG-1), and then analyzed with probes for the mutation. The experiment was performed both on preparations of fresh cells grown directly on microscopy slides, and on sections of formalin fixed and paraffin embedded cells. Samples with a ratio of as low as 1% *KRAS* mutant cells in an otherwise wild-type cell background were successfully detected (Supplementary Note 3). The effect of FFPE preparation and the importance of spatial context are also discussed in Supplementary Note 3.

### Expression patterns in tumors with multiple mutations

**Figure 6:**
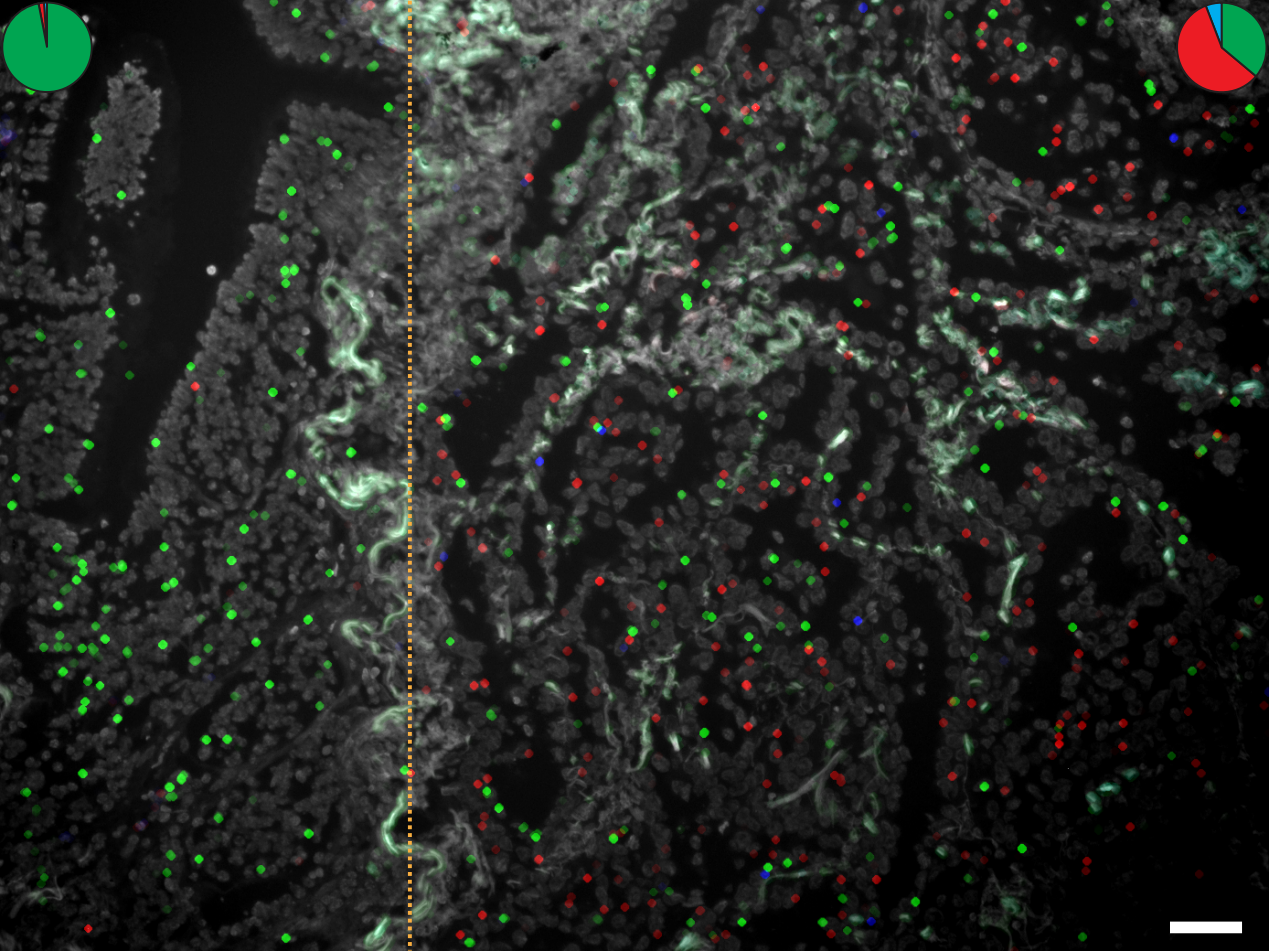
Detection of two *EGFR* point mutations, G719C and S768I, using mutation-specific padlock probes giving rise to differently colored RCPs; green RCPs represent wild-type *EGFR*, red RCPs G719C mutants and blue RCPs S768I mutants Cell nuclei are shown in grey. The left part of the tissue represents benign respiratory epithelium of a small bronchus that expresses wild-type *EGFR* whereas the right part shows a representative tumor area with cancer cells displaying RCPs from the *EGFR* wild-type, G719C and S768I padlock probes. The pie charts indicate the ratio between wild-type (green), G719C mutant (red) and S768I (blue) signals in two different areas of the tissue (dotted line denotes border). Scale bar, 50 μm.

To further study intratumor heterogeneity, we designed probes for tumors that were known to harbor multiple point mutations. As a proof-of-concept that intratumor heterogeneity can be studied, we established individualized *in situ* mutation assays for screening of FFPE cases carrying unique combinations of mutations in *EGFR*, *KRAS*, and *TP53* (Table 1). One lung cancer case was positive for the activating *EGFR* mutation G719C, as well as the *EGFR* S768I mutation that is associated with resistance to anti-EGFR therapy (Fig. 6 and Supplementary Fig. 11A). The expression of the G719C transcript was high compared to the S768I transcript throughout the tumor section. This balance between the expressed mutated alleles might be expected as this case represents a patient that had not received anti-EGFR therapy so no selection pressure for increased expression of the resistance mutation was present.

**Figure 7:**
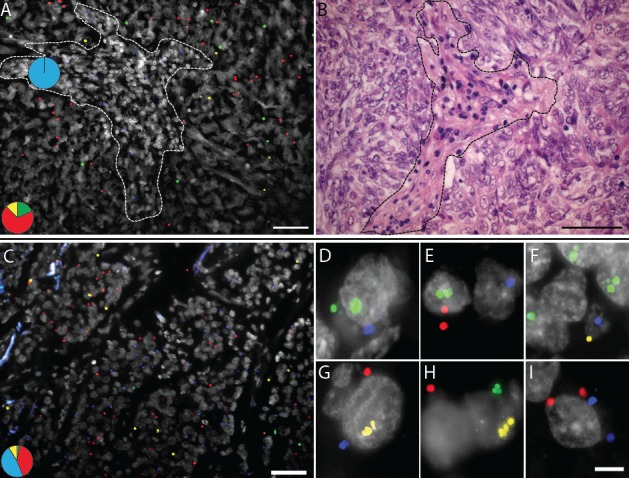
*In situ* mutation analysis of FFPE lung tumor samples known to harbor (A, B) an *EGFR* G719A and a *TP53* S127F mutation or (C-I) a reported *KRAS* G12C and a *TP53* P190S mutation (A) Green RCPs represent wild-type *EGFR*, yellow RCPs mutant *EGFR* G719A, blue RCPs wild-type *TP53* and red RCPs represent mutant *TP53* S127F. The image represent cells in stromal regions (dotted line) in which only the wild-type form of *TP53* is expressed while the tumor regions displayed RCPs originating from the S127F padlock probe as well as the wild-type *EGFR* and G719A padlock probes. The pie charts indicate the ratio between wild-type *EGFR* (green), *EGFR* G719A mutant (yellow), wild-type *TP53* (blue) and mutant *TP53* S127F (red) signals in two different areas of the tissue (dotted line denotes border). Cell nuclei are shown in grey. (B) Brightfield image from the same tumor region in a consecutive H&E-stained section. Scale bars, 50 μm. (C) Merged image showing a homogenous expression pattern in a tumor area of mutated and wild-type *TP53* and *KRAS* alleles. Green RCPs represent wild-type *KRAS*, yellow RCPs mutant *KRAS* G12C, blue RCPs wild-type *TP53* and red RCPs represent mutant *TP53* P190S. The pie chart indicates the ratio between wild-type *KRAS* (green), *KRAS* G12C mutant (yellow), wild-type *TP53* (blue) and mutant *TP53* P190S (red) signals in the tissue. Cell nuclei are shown in grey. Scale bar, 50 μm. (D-I) Panel of single cells displaying different signal patterns zoomed in from the same FFPE lung sample case. (D) Wild-type *KRAS*, wild-type *TP53*, (E) wild-type *KRAS*, mutant *TP53*, (F) mutant *KRAS*, wild-type *TP53*, (G) mutant *KRAS*, wild-type and mutant *TP53*, (H) wild-type and mutant *KRAS* and (I) wild-type and mutant *TP53*. Scale bar, 5 μm.

A second lung cancer FFPE sample was assayed for a G719A *EGFR* mutation in combination with a S127F mutation of the tumor suppressor gene *TP53* (Fig. 7A, B and Supplementary Fig. 11B). The *in situ* analysis showed cells in stromal regions that only expressed the wild-type form of *TP53* while no expression of any of the *EGFR* alleles could be detected. H&E staining of this tissue sample confirmed that the cell populations with wild-type *TP53* were lymphocytes. The *TP53* S127F mutation-positive tumor regions displayed signals from both the wild-type *EGFR* and G719A padlock probes but none from the wild-type *TP53* padlock probe, indicating *TP53* LOH.

Finally, we applied a set of padlock probes on a FFPE lung tissue sample with reported *KRAS* G12C and *TP53* P190S mutations. In contrast to the previous case, in which the wild-type and mutant *TP53* signals were located in different compartments (stroma and tumor respectively), here the mutant and wild-type *TP53* transcripts were expressed in a heterozygous fashion in the tumor compartment (Fig. 7C). Similarly, the wild-type and mutant *KRAS* signals were evenly distributed across the tumor areas with a higher expression of mutant compared to wild-type *KRAS* alleles. This difference in expression pattern of the wild-type and mutant alleles in the two cases would not have been identified unless an *in situ* technique was included as a complement to DNA sequencing. Moreover, since this *in situ* assay reveals information on a single cell level, unique information (e.g. expression of more than one mutation in the same cell (Fig. 7D-I)), can be identified and studied in detail. Due to the relatively low density of signals for these transcripts in this tissue, such co-occurrences are relatively rare in our data. However, co-localization of different alleles in the same cell provides strong evidence of their co-existence in cells in the tumor while absence of co-localization does not prove that they are not co-expressed in a certain cell-lineage. Even though all four alleles were not detected in any of these cells, some cells displayed mutations in both *KRAS* and *TP53* and the most likely interpretation of the staining pattern in Fig. 7C-I is that the *KRAS* mutation is carried by all *TP53* mutation-positive cells.

## DISCUSSION

We here report the establishment of a multiplex *in situ* assay that specifically targets point mutations on tumor tissue sections and on cytological preparations. cDNA copies of transcripts, synthesized by reverse transcription of mRNA *in situ*, are targeted with mutant- or wild-type specific padlock probes and amplified to a detectable level with RCA. The resulting wild-type and mutated products are thereafter labeled with fluorophores of different colors.

To our knowledge, this padlock probe-based assay demonstrate for the first time that mutation analysis for molecular cancer diagnostics can be performed directly on tumor tissue sections. As a proof-of-concept we developed and validated a multiplexed *in situ* assay for the activating point mutations in *KRAS* codon 12 and 13 that are associated with resistance to anti-EGFR therapy in colorectal cancer. First, the selectivity of the probes was tested individually, and there was a clear-cut distinction between the *KRAS* mutant and wild-type samples and the genotypes were easily determined by simple microscopic visualization of the corresponding fluorescent signals. Second, we used unfixed tumor cells on touch imprints from the fresh cut tumor surface, and demonstrated that a validated *KRAS* mutation status could be obtained on the day of sample arrival. This could potentially be extended to analyses of other cytological preparations, such as smears after fine needle aspiration or immobilized circulating tumor cells, using the same protocol. Third, to enable routine clinical applications of the technology, we developed a procedure for mutational analyses in FFPE tissue sections.

Paraffin-embedded tissue blocks are used globally in routine surgical pathology and can be preserved for years in tissue archives. However, crosslinking of biomolecules induced by formalin results in fragmentation of DNA and RNA. The short length of the padlock probe, in combination with the requirement of dual recognition sites and ligation makes this assay ideal for fixed histopathology specimens. Using a protocol optimized for formalin-fixed tissues we achieved *in situ* mutation detection in routine FFPE sections, and prospective surgical cancer specimens with unknown *KRAS* status were successfully characterized. A promising prospect for this assay is that hundreds of FFPE cancer samples can be screened simultaneously in TMAs for presence of mutations. Thus, for biomarker discovery in retrospective patient cohorts with available TMAs, high-throughput screening for point mutations could be performed along with IHC for protein expression and FISH-analysis for chromosomal aberrations [16].

Despite the fact that the 84 patient samples (10 fresh frozen, 8 touch imprints and 66 FFPE tissues) assayed in this study had been collected at different time points during the last two decades and represented many different types of clinically relevant tissue material with different degree of RNA preservation, they all proved to qualify as suitable tissue material. The samples were not selected on a priori knowledge about RNA preservation, and they were not quality tested before analysis. All samples that entered the study are presented in this study, none were excluded. Furthermore, padlock probes were successfully applied for *in situ* detection of totally 14 different point mutations which have made us confident that our mutation assay offers robustness and can easily be adapted for detection of other mutations on tissue material from various sources.

Our *in situ* RNA detection approach yields data that can be quantified by digital spot counting, and has previously been shown to correlate well with the RNA content of fixed cells from cell cultures as determined by quantitative PCR (qPCR) [2]. In cell lines, the detection efficiency (i.e. the fraction of mRNA molecules present in the cell that is detected) has been estimated to 30%. In tissue sections, this number is probably lower due to loss and degradation of RNA upon preservation, storage, sectioning, and slide preparation. The quality of sections will vary from tissue to tissue, and perhaps most prominently in FFPE tissues. The loss of RNA due to issues with preservation is likely to affect different mRNAs at similar rate, so the quality of the tissue section can be assessed by *ACTB* or similar housekeeping gene staining. Indeed, we note that the density of signals varies from tissue to tissue, and in some FFPE sections the density of signals is quite low. However, the relative abundance of our signals for the different transcripts seems to be similar, and they agree well with published RNA level estimates in normal colon, normal lung, and lung cancer tissue (Supplementary Note 4) [17].

The sensitivity of mutation assays based on DNA-extraction in combination with sequencing or allele-specific PCR is directly linked to the tumor cell content of the sample. In contrast, the padlock probe-based RCA method in principle, as other *in situ* assays such as IHC or FISH, is independent of the relative tumor cell fraction, as a correct mutation score will rely on the microscopical identification of a representative group of tumor cells with a sufficient number of specific signals. In order to define the practical sensitivity in a clinical setting, a side-by-side comparison with a standard diagnostic mutation test needs to be performed in a large consecutive patient cohort. However, as mutations in a wide range of sample types were correctly scored in the present study, including core needle biopsies with a tumor cell fraction as low as 10% and even lower in many of the TMA cores, we are confident that this assay will perform in tissue materials relevant to routine cancer diagnostics.

Padlock probes display very little cross-reactivity [18] and could thus be used as a general mutation analysis tool with multiplex detection of a set of relevant cancer targets. The only known limitation is the number of available fluorophores to separate the targets from each other. An alternative is therefore to design padlock probes with many detection sites and identify the targets sequentially [19], or by in situ sequencing [20]. The *in situ* protocol can be adapted for automation as any conventional FISH-assay, facilitating implementation of the assay for routine use. The fluorescence readout can also be changed to a histochemical staining for brightfield imaging if desired, similar to what was done in a recent *in situ* RCA-approach [21].

The concept of tumor heterogeneity is complex with one aspect being the variable content of cancer cells with acquired somatic mutations and genetically normal stromal and inflammatory cells. A second aspect is the morphological, and possibly genetic, variation within the tumor compartment with regard to pre-neoplastic versus invasive components, high-grade versus low-grade areas, invasion front versus central tumor area, and variable differentiation patterns. A third aspect is that the expression of a mutated allele can be influenced by promoter and splicing mutations, epigenetic alteration, or gene copy number aberrations, e.g. amplifications, deletions and LOH, in different parts of the tumor. These may be challenging to analyze on a genomic level, but they will in many instances be evident on an mRNA level. Loss of expression of tumor suppressor genes due to bi-allelic deletions, nonsense mediated decay or promoter methylation can be detected as loss of signals compared to a housekeeping gene, but such analysis will probably be less sensitive than the detection of gain of function alleles, where just a few signals will be enough to score positivity. The described *in situ* technique allows studies of all these features of intratumor heterogeneity.

Heterozygous and homozygous expression of mutated and wild-type alleles can be appreciated in tumor cells and demonstrate one form of fundamental information about a particular tissue specimen that probably would have gone undetected with PCR-based techniques resulting in an average value of the extracted mixture of mutant tumor and wild-type cells. We show increased expression of a mutated *KRAS* codon 61 allele along with tumor progression in a colon cancer sample (Fig. 4). In a case of lung adenocarcinoma, the expression of an activating *EGFR* mutation was demonstrated to differ between areas with distinctive histological patterns (Fig. 5). The technique allows dissection of how multiple different mutations are distributed across a tumor lesion, as illustrated by two lung cancer cases where mutated *TP53* alleles could be visualized together with activating mutations in *EGFR* and *KRAS* respectively (Fig. 7). Thus, mutation analysis *in situ* can help to dissect processes such as cancer initiation, tumor progression and metastasis. An intriguing application will be studies of resistance mutations after targeted therapy [22]. Here we present a case with a double mutation in *EGFR* where low expression of the resistance mutation was seen in parallel with expression of the mutation associated with treatment response, as might be expected in a patient with a de novo resistance mutation (Fig. 6). Analysis of a follow-up sample after anti-EGFR treatment could reveal a patient-specific response on a histological level regarding the expression of the two mutations.

As opposed to the heterogeneity with regard to expression levels in cancer cells we could not detect any isolated clones in the sections with distinct mutation patterns reading *KRAS*, *EGFR* and *TP53* status. This is perhaps not surprising as these mutations can be expected to arise early during tumor development and thus be universally expressed. However, in future studies the *in situ* technique could be used, instead of laser microdissection, to explore late branching events during tumor evolution, such as the recently described emergence of *KRAS* mutated clones during anti-EGFR treatment of *KRAS* wild-type colorectal carcinoma [23], expansion of clones with resistance mutations in *EGFR*-treated lung carcinoma [24], and development of secondary mutations during mTOR (mammalian target of rapamycin) inhibition in renal cell carcinoma [25].

In conclusion, we believe that the presented padlock probe and RCA technology will be an important assay for at least three distinct types of applications in cancer research and diagnostics. First, it can serve as a very useful complement to next-generation sequencing for studies of histologic-genotypic correlations in complex tumor tissues and to elucidate intra-tumoral genetic heterogeneity identified by sequencing. Second, it can be used for high-throughput targeted mutation screening in TMAs and biopsy sections, and finally for diagnostic molecular pathology, directly combining histological examination and molecular diagnostics. All these applications are important aspects of translational biomarker research and development of companion diagnostics.

## MATERIALS AND METHODS

### *In situ* mutation detection on cell lines and tissues

Supplementary Figure 13 is providing detailed experimental protocols for the different tissue types and description of the sample pretreatments (Supplementary Note 1). All the molecular *in situ* reactions were carried out in Secure-seals (Grace Bio-Labs Inc.) and the reaction volumes for tissues or imprints were either 100 μl (size 13 mm diameter, 0.8 mm deep) or 350 μl (size 22 mm diameter, 0.8 mm deep) depending on the size of the sample. The Secure-seals that were used for cells had a total volume of 50 μl (size 9 mm diameter, 0.8 mm deep). The Secure-Seals were mounted over the cells or tissues and the wells were dehydrated by a brief flush with PBS-T (DEPC-PBS with 0.05% Tween-20 (Sigma)). Postfixation of fresh frozen and FFPE tissues was performed for 45 min compared to 30 min for cell lines and tumor imprints. Also, for postfixation of FFPE tissues 3.7% formaldehyde was used instead of 3% (w/v) paraformaldehyde. Finally, the RCA incubation time performed on tissues was longer (5 h) compared to cultured cells and tumor imprints (2 h). For all reactions slides were incubated in humid chambers.

### Primers, padlock probes and detection probes

Oligonucleotide sequences (Supplementary Table 1) were designed using GenBank accession numbers NM_033360 (*KRAS*), NM_005228 (*EGFR*), NM_001126114.1 (*TP53*) and NM_001101.3 (*ACTB*). All padlock probes were 5' phosphorylated at a concentration of 10 μM with 0.2 U μl^−1^ T4 PNK (Fermentas) in PNK buffer A and 1 mM ATP for 30 min at 37 °C, followed by 10 min at 65 °C. The primers, padlock probes and detection probes applied on the different tissue samples and cell lines are summarized in Supplementary Material and shows mutant padlock probes and their distribution of mutant and/or wild-type signals in the tested samples (Supplementary Table 2 and Supplementary Table 3).

### *In situ* mutation detection

One μM of cDNA primer was added to the slides with 20 U μl^−1^ of RevertAid H minus M-MuLV reverse transcriptase (Fermentas), 500 μM dNTP (Fermentas), 0.2 μg μl^−1^ BSA (NEB), and 1 U μl^−1^ RiboLock RNase Inhibitor (Fermentas) in the M-MuLV reaction buffer. Slides were incubated for 3 hours at 37 °C. After incubation slides were washed briefly by flushing the wells in PBS-T, followed by a postfixation step for 45 (fresh frozen and FFPE tissues) or 30 (imprints) min at room temperature. After postfixation the samples were washed by flushing the Secure-seals chambers with PBS-T.

To create single-stranded target cDNA available for padlock probe hybridization, the RNA part of the created RNA-DNA hybrids was degraded with RNase H. This was performed in the same step as hybridization and ligation of the padlock probes. The reaction was carried out with 100 nM of each padlock probe in a mix of 1 U μl^−1^ Ampligase (Epicentre), 0.4 U μl^−1^ RNase H (Fermentas), 1 U μl^−1^ RiboLock RNase Inhibitor, 50 mM KCl, 20% formamide in Ampligase buffer. Incubation was performed first at 37 °C for 30 min, followed by 45 min at 45 °C. After ligation, slides were washed by flushing the chambers with PBS-T. For prospective *KRAS* mutation detection of unknown tissue samples a cocktail of all *KRAS* codon 12 and 13 padlock probes was mixed with a final concentration of 10 nM.

RCA was performed with 1 U μl^−1^ Φ29 DNA polymerase (Fermentas) in the supplied reaction buffer with 1 U μl^−1^ RiboLock RNase Inhibitor, 250 μM dNTP, 0.2 μg μl^−1^ BSA, and 5% glycerol. Incubation was carried out for 2 h for tumor imprints as well as for cell lines and approximately 5 h for fresh frozen and FFPE tissues at 37 °C. After RCA the samples were washed by flushing the Secure-seals chambers with PBS-T. RCPs were visualized using 100 nM of each corresponding detection probe in 2× SSC and 20% formamide at 37 °C for 15 min. Slides were then washed again by flushing the chambers in PBS-T, the Secure-seals were removed and the slides were dehydrated using a series of 70%, 85%, and 99.5% ethanol for 30 sec each. The dry slides were mounted with Vectashield (Vector), containing 100 ng ml^−1^ DAPI to counterstain the cell nuclei.

### Image analysis and acquisition

The mutation status in the tested samples was based on visual examination using an AxioplanII epifluorescence microscope (Zeiss), equipped with a 100 W mercury lamp, a CCD camera (C4742-95, Hamamatsu), and a computer-controlled filter wheel with excitation and emission filters for visualization of DAPI, FITC, Cy3, Texas Red and Cy5. The tissue sections were carefully analyzed to ensure correct scoring of mutation in representative tumor areas predefined by histopathological examination of corresponding H&E-stained sections. The *in situ* scoring procedure of wild-type or mutant tumors is further discussed in Supplementary Material (Supplementary Note 2).

For capturing the images, a ×10 (Plan-Apocromat, Zeiss) and a ×20 (Plan-Apocromat, Zeiss) objectives were used for fresh frozen and FFPE tissues, a ×20 objective for tumor imprints and finally a ×63 (Plan-neofluar, Zeiss) objective was used for the cells. Images were collected using the Axiovision software (Release 4.8, Zeiss). Images displayed for illustrations were processed using image editing software for clarity in print. The threshold for different color channels was set using ImageJ 1.42q and for clearer visualization of the RCPs, a maximum filter was applied. Autofluorescence in some samples, e.g. lung tissues with extensive connective tissue, was reduced in the ImageJ software by subtracting background signals. For cell lines, core biopsies and imprints (Fig. 3C-F and Supplementary Fig. 1, 8 and 10J-N), a maximum intensity projection was created in Axiovision using the collected *z*-stack images.

Quantification of signals/image was performed manually for figure 1-7 and the results are presented as pie charts illustrating the ratio between the differently colored signals in respective image.

## Supplementary Notes, Tables and Figures


