# Supplementary material for: In situ mutation detection and visualization of intratumor heterogeneity for cancer research and diagnostics
Source: Oncotarget. 2013 Nov 21;4(12):2407–18. doi: 10.18632/oncotarget.1527 (PMC3926836; doi:10.18632/oncotarget.1527)
Supplement: Supplementary file 1 [file oncotarget-04-2407-s001.docx]

***In situ* mutation detection and visualization of intratumor heterogeneity for cancer research and diagnostics**

Ida Grundberg, Sara Kiflemariam, Marco Mignardi, Juliana Imgenberg-Kreuz, Karolina Edlund, Patrick Micke, Magnus Sundström, Tobias Sjöblom, Johan Botling^*^ and Mats Nilsson^*^

*Authors contributed equally

**Supplementary Material:**

| **Supplementary Figure 1** | *In situ KRAS* mutation detection in cell lines. |
| --- | --- |
| **Supplementary Figure 2** | *In situ KRAS* mutation detection in fresh frozen colon samples. |
| **Supplementary Figure 3** | *In situ KRAS* mutation detection in fresh frozen lung samples. |
| **Supplementary Figure 4** | Comparison between single- and multiplex *in situ* mutation detection. |
| **Supplementary Figure 5** | *KRAS* mutation detection in FFPE colon samples. |
| **Supplementary Figure 6** | Detection of Q61H *KRAS* point mutation in FFPE colon samples. |
| **Supplementary Figure 7** | Multiplex detection of *KRAS* mutations in prospective FFPE lung samples. |
| **Supplementary Figure 8** | Multiplex detection of *KRAS* mutations in prospective colon tumor imprints. |
| **Supplementary Figure 9** | Multiplex detection of *KRAS* mutations in prospective FFPE colon TMA. |
| **Supplementary Figure 10** | Detection of the L858R point mutation of *EGFR* in FFPE lung samples. |
| **Supplementary Figure 11** | *In situ* detection of multiple mutations in FFPE samples. |
| **Supplementary Figure 12** | Pyrosequencing data. |
| **Supplementary Figure 13** | Flowcharts experimental protocols. |
| **Supplementary Table 1** | Oligonucleotide sequences. |
| **Supplementary Table 2** | Oligonucleotides applied on tissue samples. |
| **Supplementary Table 3** | Padlock probe distribution on samples. |
| **Supplementary Note 1** | Sample pretreatment |
| **Supplementary Note 2** | Tissue classification and scoring of mutations. |
| **Supplementary Note 3** | Assay sensitivity by serial cell dilution experiment. |
| **Supplementary Note 4** | Comparison with published expression levels in relevant tissues. |

**Supplementary Figure 1**


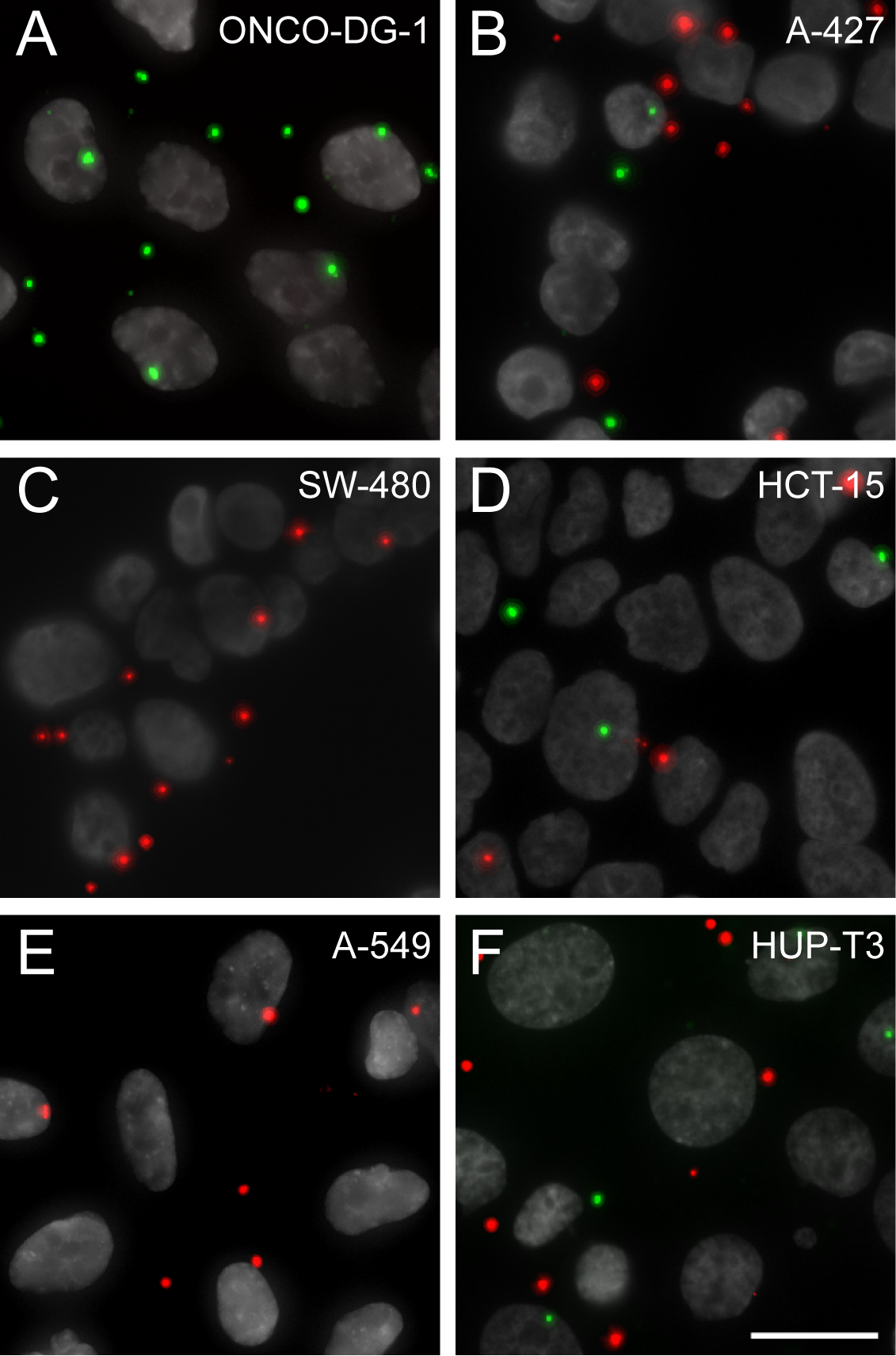


**Supplementary Figure 1:** ***In situ* transcript detection of *KRAS* codon 12 and 13 point mutations on mutation specific cell lines using padlock probes and target-primed RCA.** Detection of *KRAS* wild-type (green RCPs) and mutants (red RCPs) in (A) the wild-type cell line ONCO-DG-1, (B) the heterozygous mutant cell line A-427 (G12D), (C) the homozygous mutant cell line SW-480 (G12V), (D) the heterozygous mutant cell line HCT-15 (G13D), (E) the homozygous mutant cell line A-549 (G12S) and (F) the heterozygous mutant cell line HUP-T3 (G12R). Cell nuclei are shown in grey. Scale bar, 20 µm.

**Supplementary Figure 2**


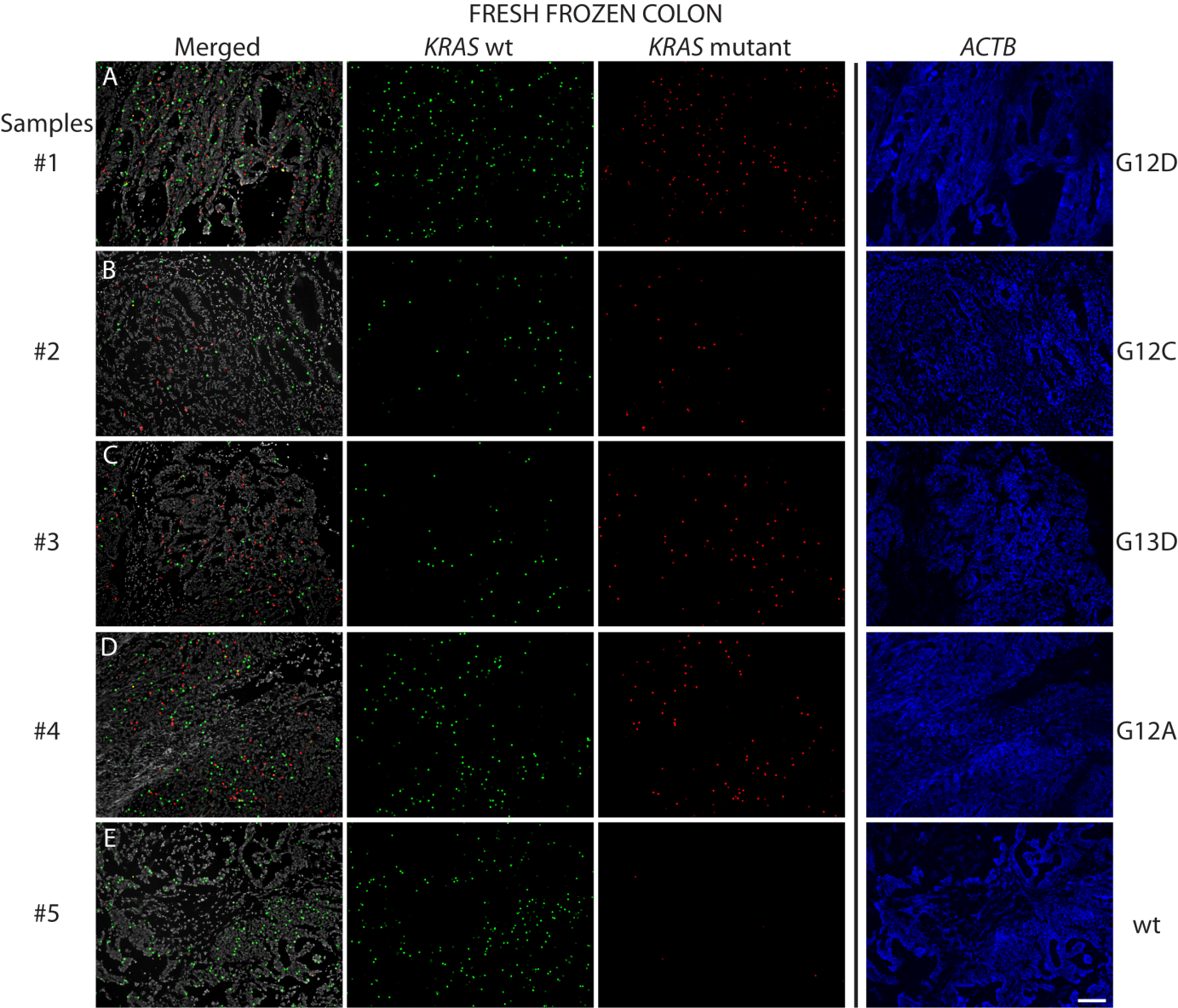


**Supplementary Figure 2: Panel of all fresh frozen colon cancer tissue sections, with known *KRAS* status, that were analyzed *in situ* with padlock probes and RCA.** The numbers (#1-5) represent the case numbers in **Table 1**. The samples correspond to (A) G12D, (B) G12C, (C) G13D, (D) G12A *KRAS* mutations and (E) a *KRAS* wild-type colon tumor tissue section. Red RCPs represent mutant *KRAS* and wild-type signals are shown as green spots. The images are presented in a merged format as well as in respective color to show the distribution of the target transcripts. Also, *ACTB* was detected in the same tissues and its expression is displayed in blue. Nuclei are shown in grey. Scale bar, 10­­­­0 µm.

**Supplementary Figure 3**

**
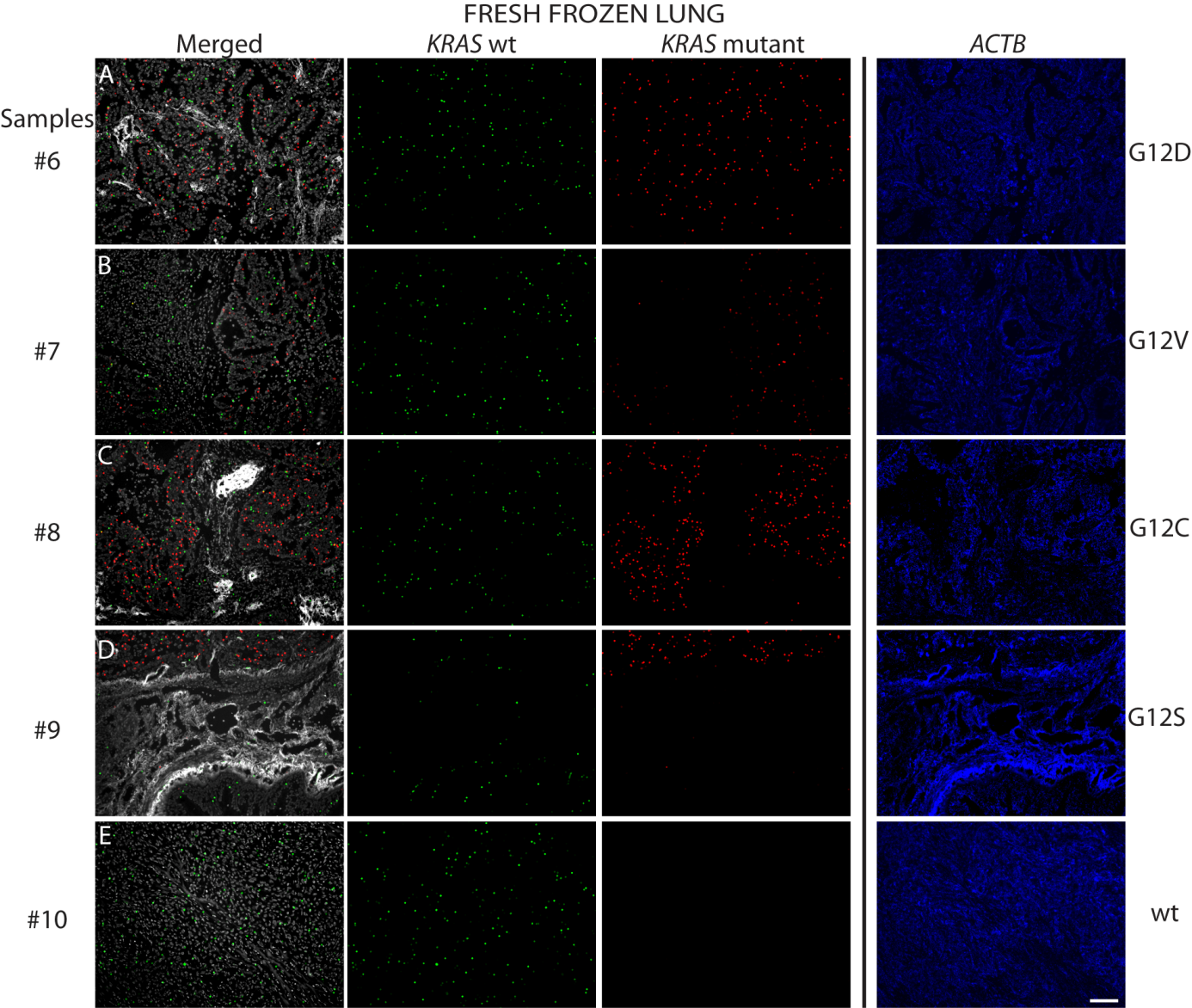
**

**Supplementary Figure 3: Panel of all fresh frozen lung cancer tissue sections, with known *KRAS* status, that were analyzed *in situ* with padlock probes and RCA.** The numbers (#6-10) correspond to the case numbers in **Table 1**. The samples represent (A) G12D, (B) G12V, (C) G12C, (D) G12S *KRAS* mutations and (E) a *KRAS* wild-type lung tumor tissue section. Red RCPs represent mutant *KRAS* and wild-type signals are shown as green spots. The images are presented in a merged format as well as in respective color to show the distribution of the target transcripts. Also, *ACTB* was targeted in the same tissues and its expression is displayed in blue. Nuclei are shown in grey. Scale bar, 10­­­­0 µm.

**Supplementary Figure 4**


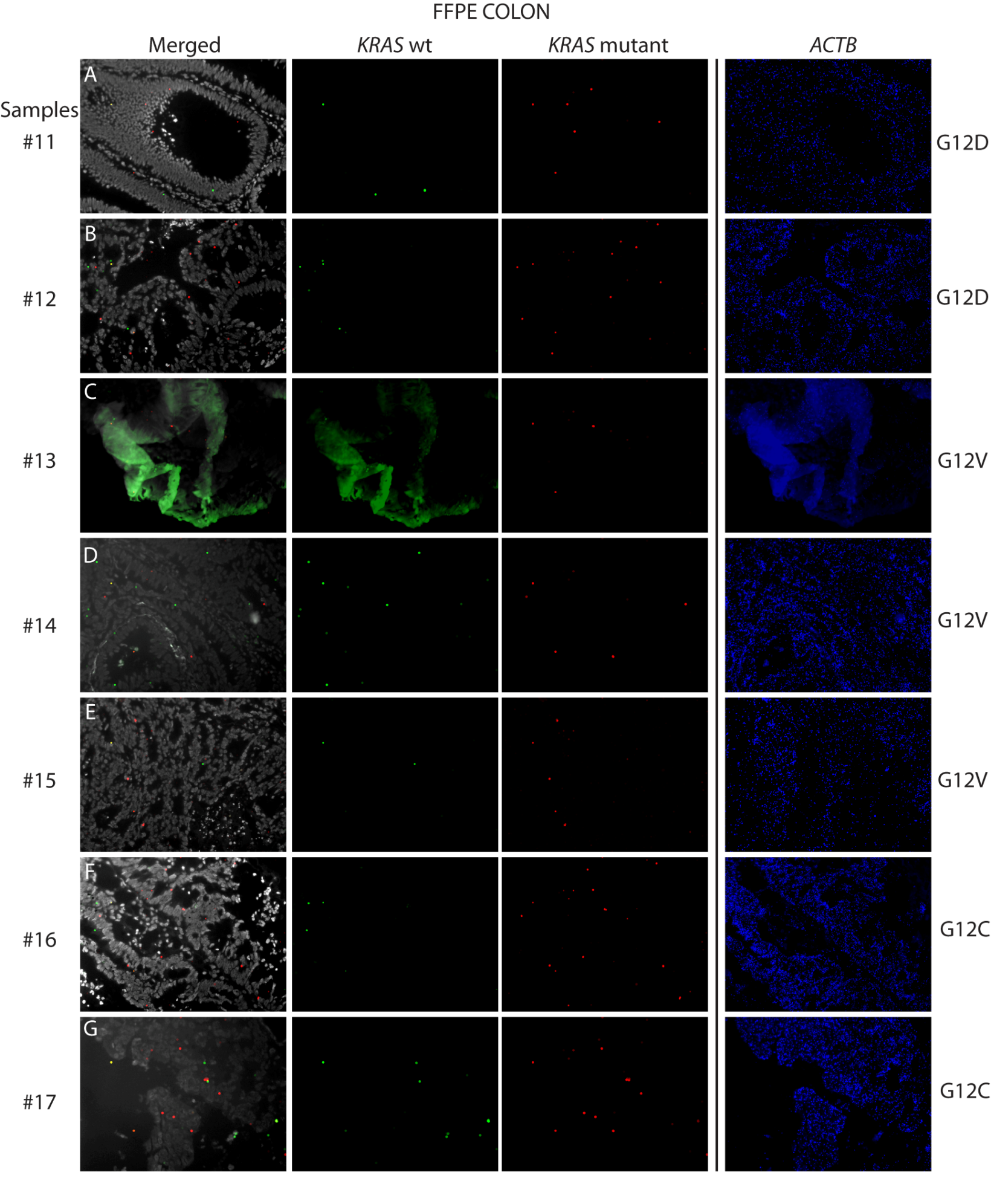

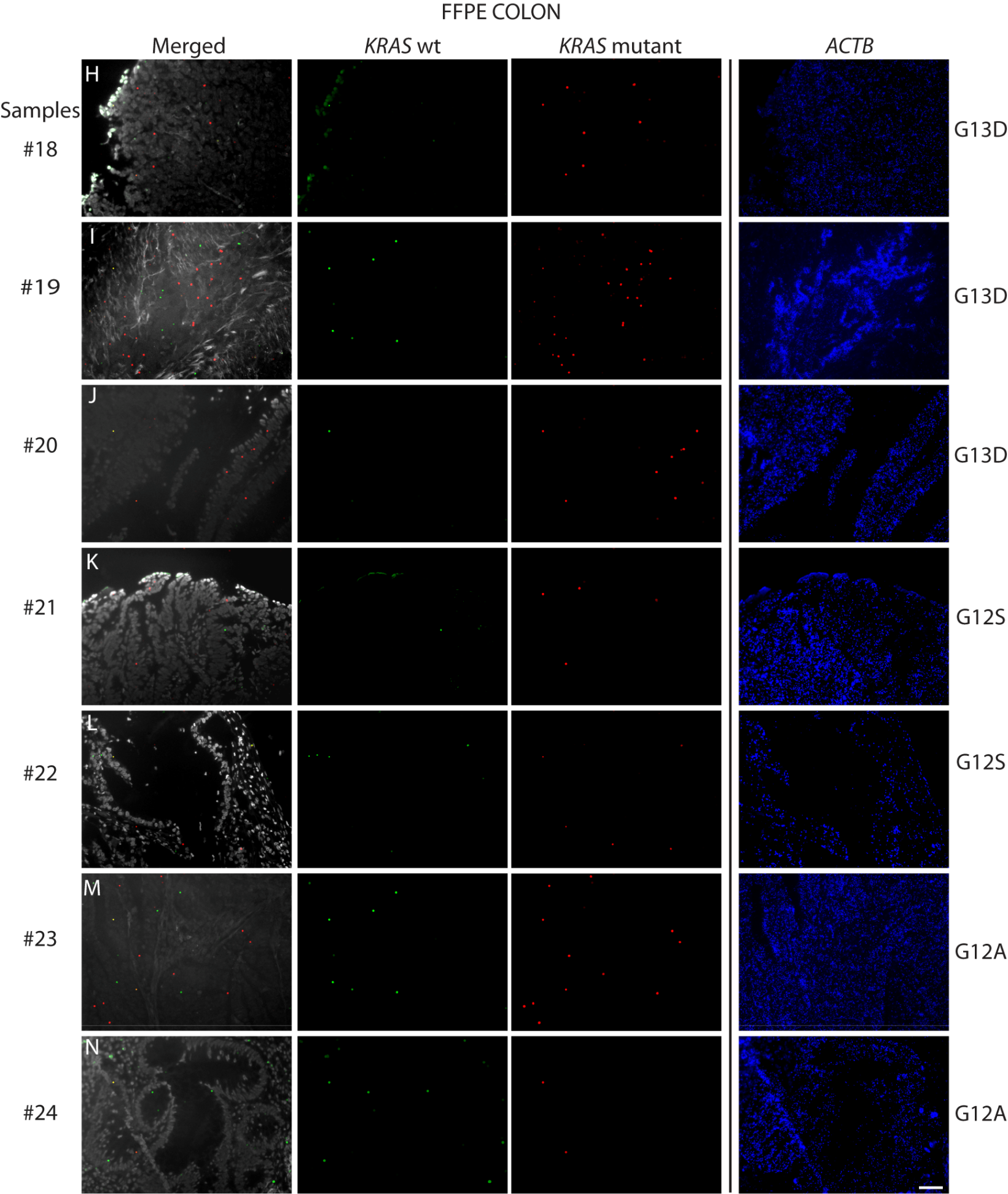
**Supplementary Figure 4: Specific *KRAS* mutation detection, using padlock probe-pairs, were applied on FFPE colon samples that represent six of the seven most common mutations reported in codon 12 and 13 of *KRAS*.** The numbers (#11-24) represent the case numbers in **Table 1**. The *in situ* assay was applied on colon tumor tissues with (A, B) G12D, (C-E) G12V, (F, G) G12C, (H-J) G13D, (K, L) G12S and finally (M, N) G12A mutations. The images are presented in a merged format as well as in respective color to show the distribution of the target transcripts. Red RCPs show mutant *KRAS* and wild-type RCPs are shown as green spots. Also, *ACTB* was targeted in the same tissues and its expression is displayed in blue. Nuclei are shown in grey. Scale bar, 5­­­­0 µm.

**Supplementary Figure 5**

**
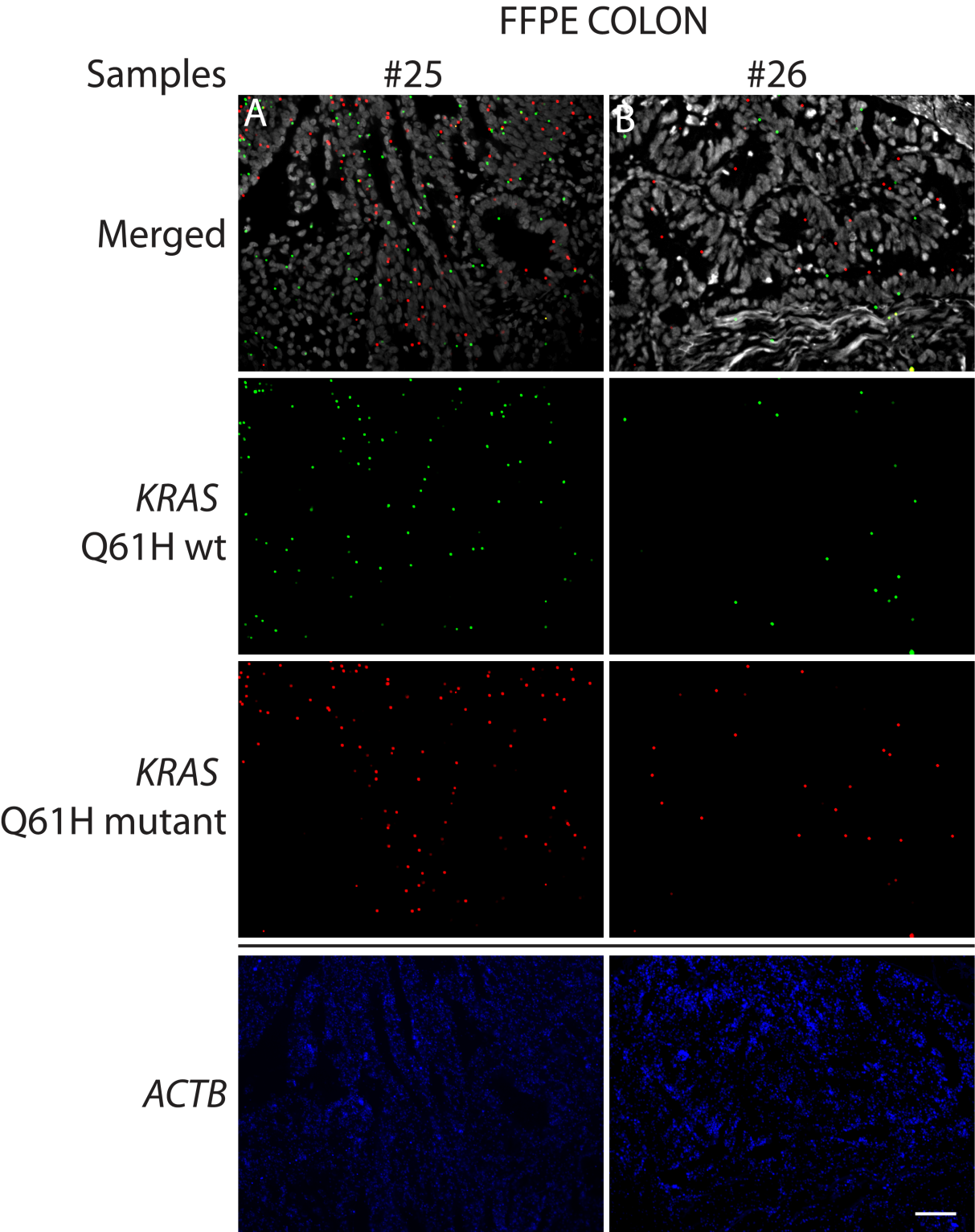
**

**Supplementary Figure 5: A padlock probe-pair, for the codon 61 Q61H *KRAS* point mutation, was tested on *(A, B)* two FFPE colon samples.** The numbers (#25-26) represent the case numbers in **Table 1.** The images are presented in a merged format as well as in respective color to show the distribution of the target transcripts. Red RCPs show mutant *KRAS* and wild-type RCPs are shown as green spots. Also, *ACTB* was detected in the same tissues and its expression is displayed in blue. Nuclei are shown in grey. Scale bar, 5­­­­0 µm. Analysis of codon 61 mutations is not performed routinely for stratification of colon cancer patients as the frequency of mutations in codon 61 of *KRAS* is low and their clinical impact with regard to EGFR inhibitor therapy is discussed. Therefore we did not establish a complete multiplex padlock probe assay for all known codon 61 mutations. However, recent studies argue that codon 61 mutations are more common than believed and indicate an association with resistance to EGFR therapy.

**Supplementary Figure 6**

**
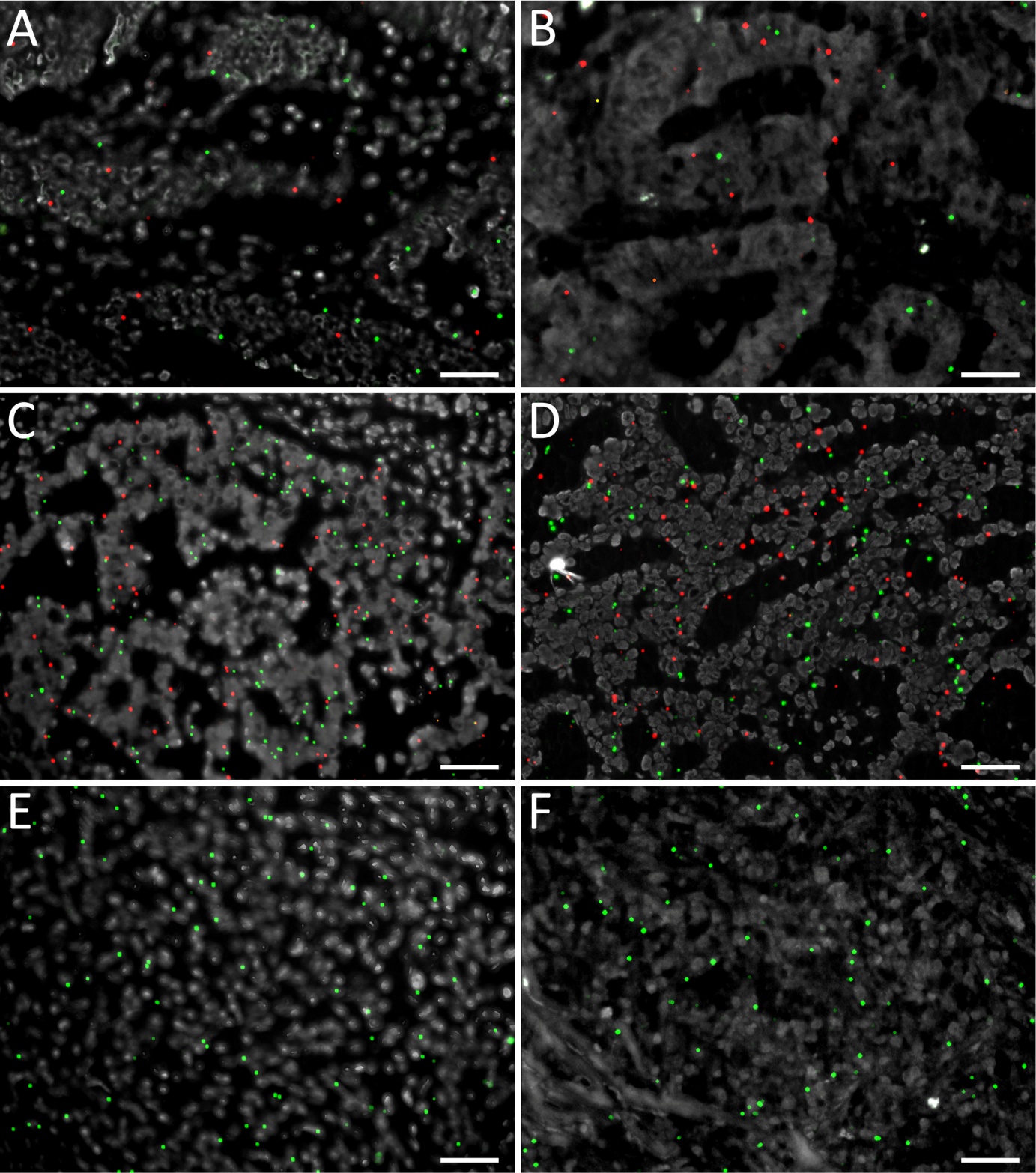
**

**Supplementary Figure 6: A comparison between (A, C, E) a singleplex mutation detection approach, using single padlock-pairs, and (B, D, F) multiplex mutation detection by applying all *KRAS* padlock probes for codon 12 and 13 on fresh frozen colon and lung tumor tissues.** The tissues display *KRAS* mutant (red) and wild-type (green) RCPs and cell nuclei are shown in grey. The two approaches were tested and compared on (A, B) a colon sample with confirmed G12D *KRAS* mutation, (C, D) a lung tissue with reported G12V *KRAS* mutation and in (E, F) a *KRAS* wild-type lung tissue. Scale bar, 50 μm.


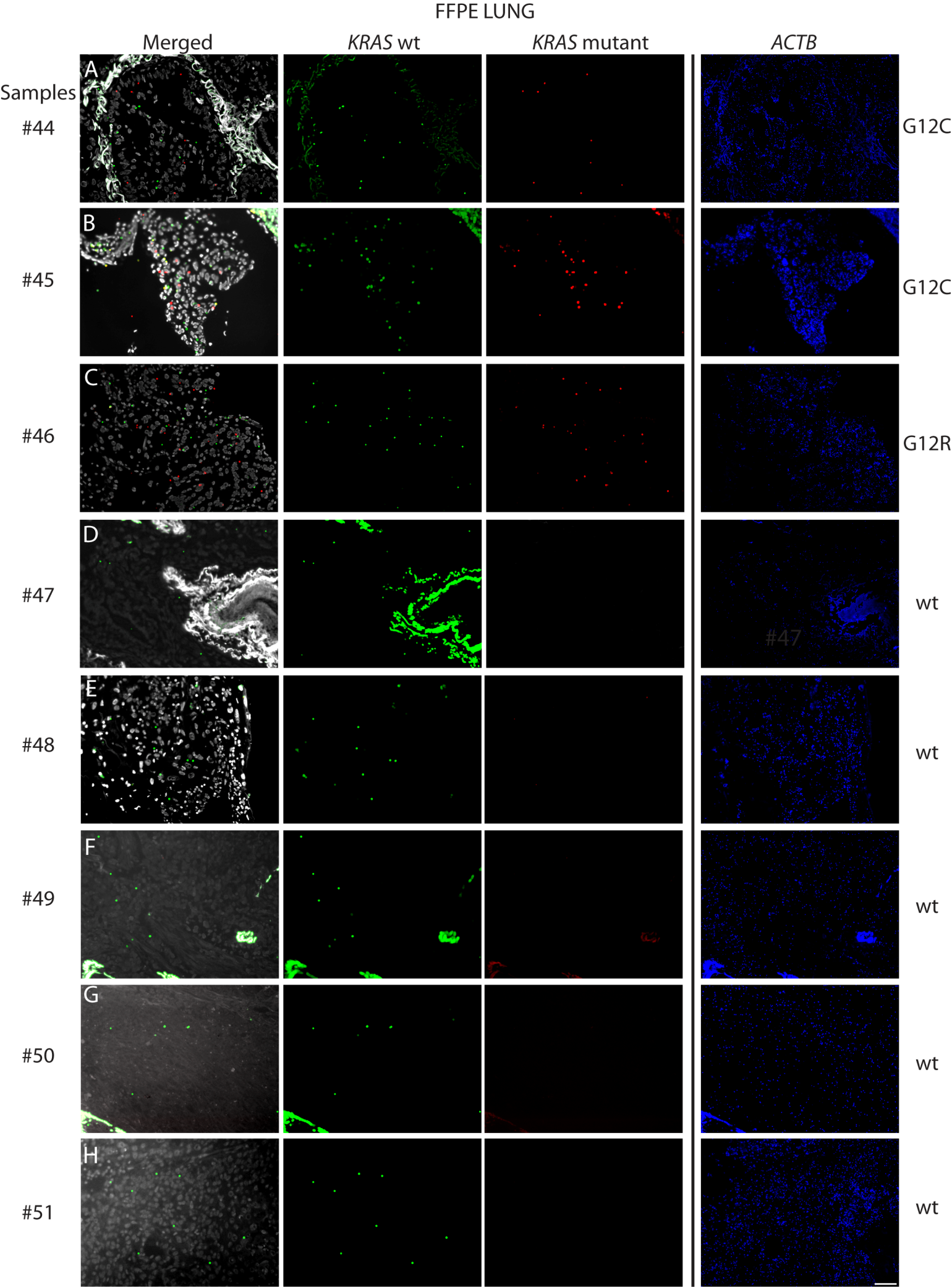
**Supplementary Figure 7**

**Supplementary Figure 7: Panel of eight FFPE lung tumor tissues with unknown *KRAS* mutation status.** The tissue sections were subject to multiplex mutation detection using a cocktail of all padlock probes that target the seven most common mutations reported in codon 12 and 13 of *KRAS*. The numbers (#44-51) represent the case numbers in **Table 2**. Out of the eight lung samples three were found to have (A-C) *KRAS* mutations, whereas the other five displayed (D-H) *KRAS* wild-type RCPs. The mutation status was confirmed by pyrosequencing and two of the mutants were reported to be (A, B) G12C, whereas the other mutant lung sample carried the rarest of the seven mutations, the (C) G12R mutation. Red spots show mutant *KRAS* and wild-type RCPs are shown as green signals. Also, *ACTB* was targeted in the same tissues and its expression is displayed in blue. The images are presented in a merged format as well as in respective color to show the distribution of the target transcripts. Nuclei are shown in grey. Scale bar, 5­­­­0 µm.


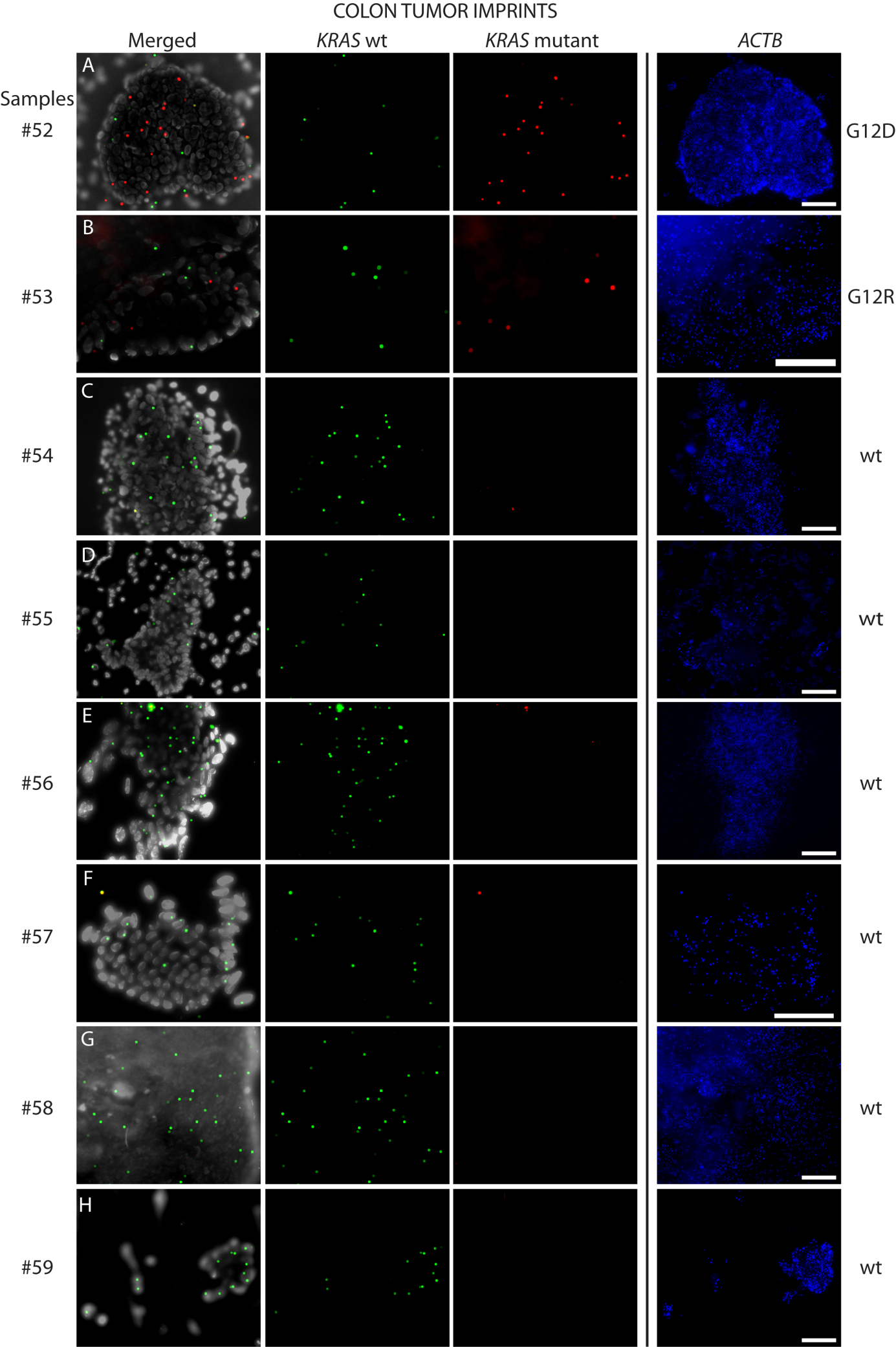
**Supplementary Figure 8**

**Supplementary Figure 8: Panel of all fresh colon touch tumor imprints that were assayed for possible *KRAS* mutations with multiplex padlock probe detection.** The numbers (#52-59) represent the case numbers in **Table 2**. The *in situ* detection analysis indicated two tumor imprints with (A, B) *KRAS* mutations and six samples with (C-H) *KRAS* wild-type tumors. Red RCPs represent mutant *KRAS* and wild-type signals are shown as green spots. Mutation status was confirmed by pyrosequencing and reported to be (A) G12D and (B) G12R mutations. The images are presented in a merged format as well as in respective color to show the distribution of the target transcripts. Also, *ACTB* was targeted in the same tissues and its expression is displayed in blue. Nuclei are shown in grey. Scale bar, 5­­­­0 µm.

**Supplementary Figure 9**


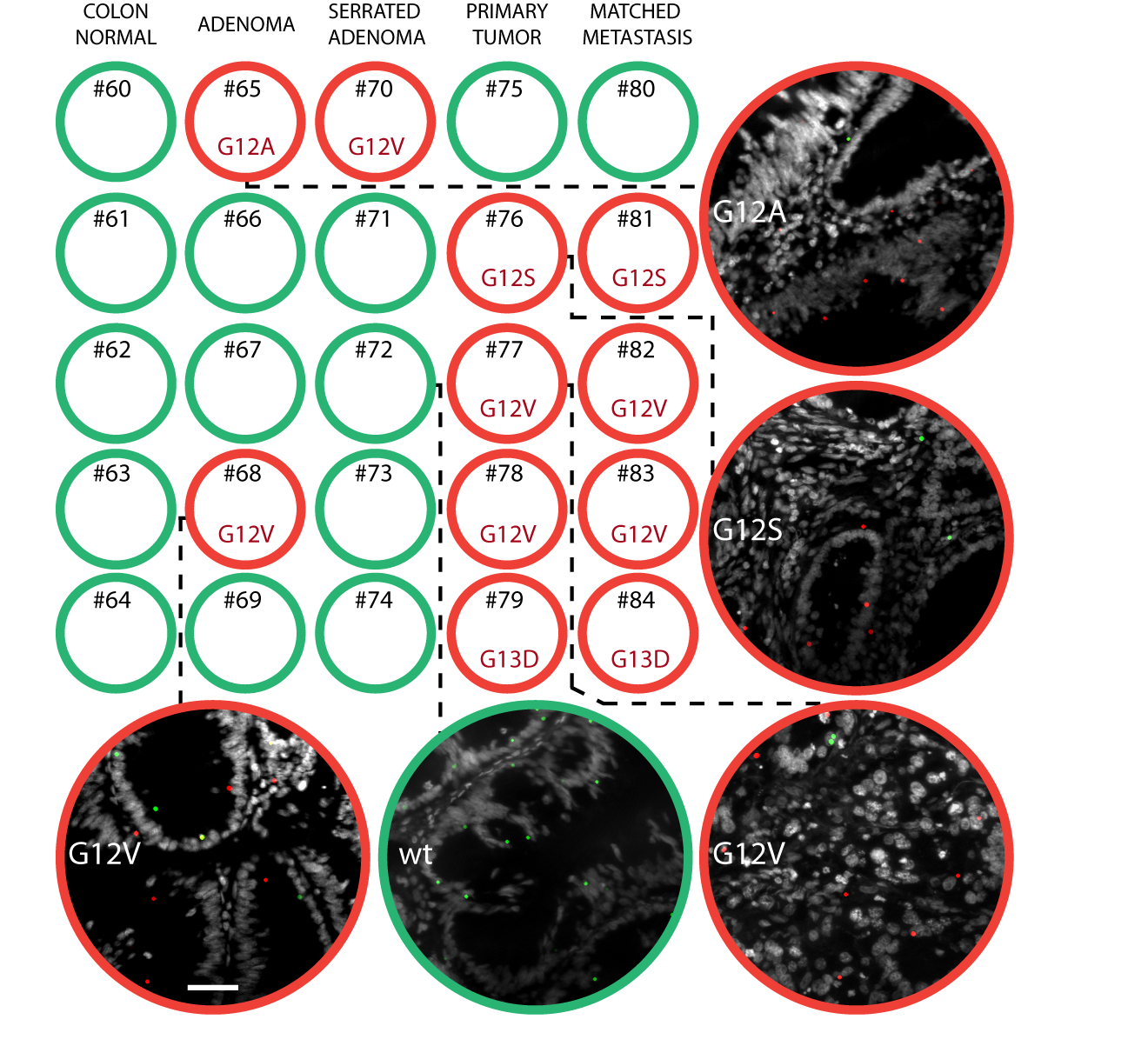


**Supplementary Figure 9: Schematic view of the FFPE colon TMA analyzed for *KRAS* codon 12 and 13 mutations.** The TMA contained tissues from normal colon, adenoma, serrated adenoma, primary tumor and matched metastasis with unknown status for *KRAS* mutations. The numbers (#60-84) represent the case numbers in **Table 2**. The *in situ* mutation analysis detected *KRAS* mutations in 11 tissues (#65, 68, 70, 76-79 and 81-84) and the remaining 14 were determined to be wild-types. Fluorescent images are showed for a selection of wild-type and mutant cases from the TMA. Red RCPs represent mutant *KRAS* and wild-type signals are shown as green spots. Nuclei are shown in grey. Mutation status was confirmed by pyrosequencing and reported to be G12A (#65), G12V (#68, 70, 77, 78, 82, 83), G12S (#76, 81) and G13D (#77, 84) mutations. Scale bar, 5­­­­0 µm.


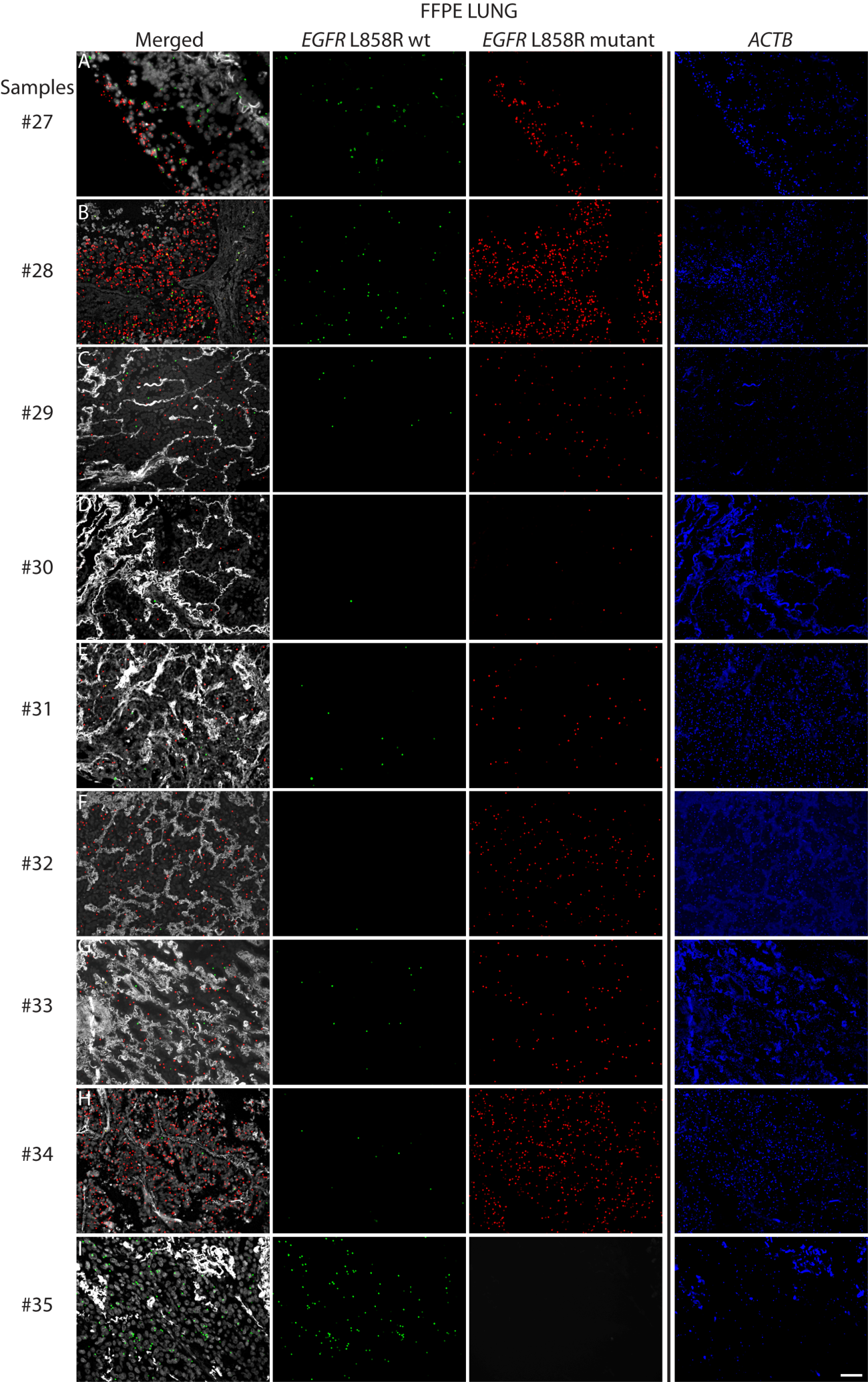
**Supplementary Figure 10**

**
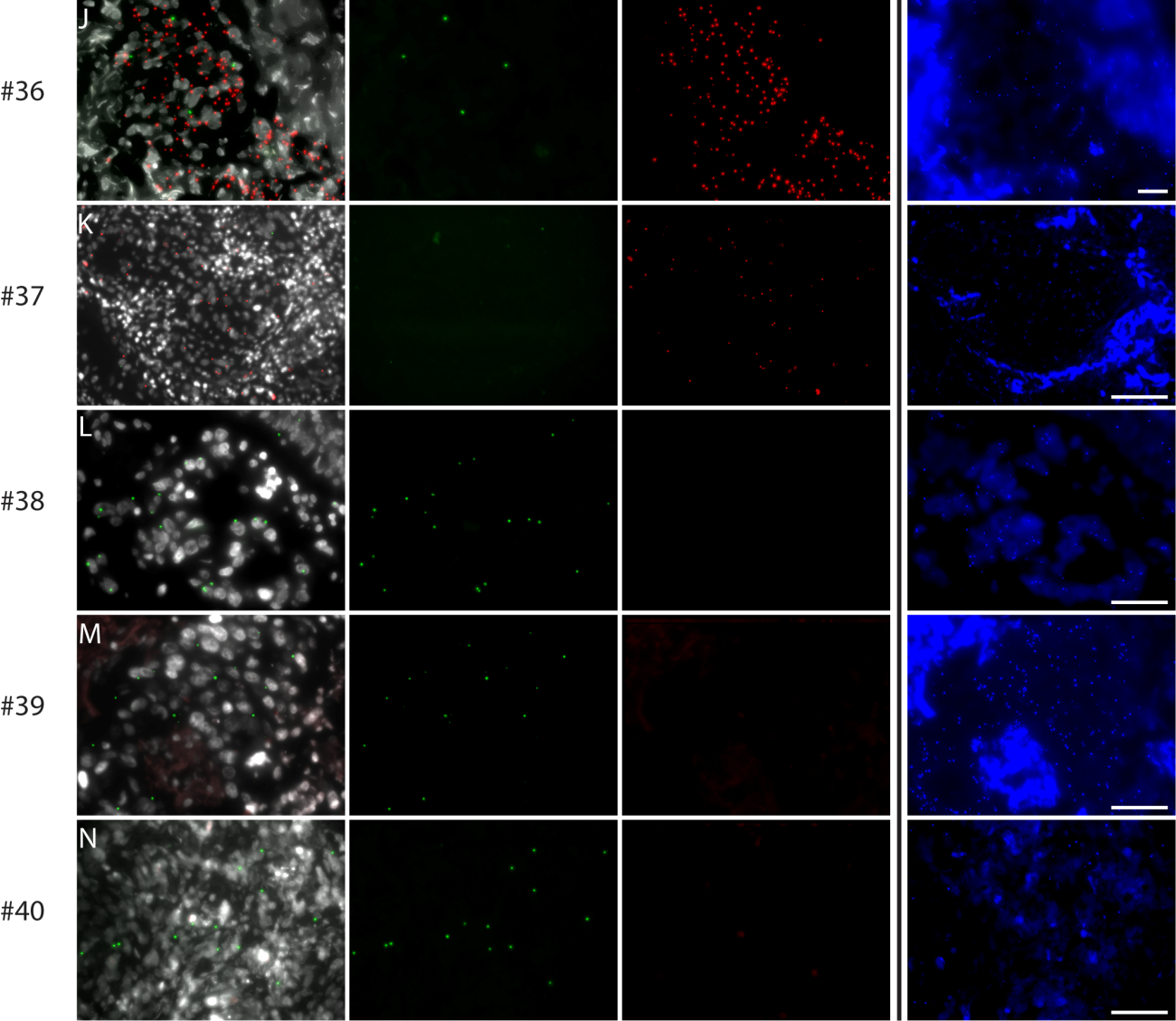
Supplementary Figure 10: Detection of the L858R point mutation of *EGFR* in lung tumor samples.** Panel of nine FFPE surgical lung tumor specimens of which eight (A-H) were positive for the L858R *EGFR* point mutation whereas the last sample (I) had wild-type status. (J-N) Five core needle biopsies from lung tumors of which two, (J, K) were known to be positive for the L858R mutation whereas the other three, (L-N) had wild-type status. The numbers (#27-40) represent the case numbers in **Table 1**. Red spots show mutant *EGFR* and wild-type RCPs are shown as green signals. Also, *ACTB* was targeted in the same tissues and its expression is displayed in blue. The images are presented in a merged format as well as in respective color to show the distribution of the target transcripts. Nuclei are shown in grey. Scale bar, 5­­­­0 µm.

**Supplementary Figure 11**

**
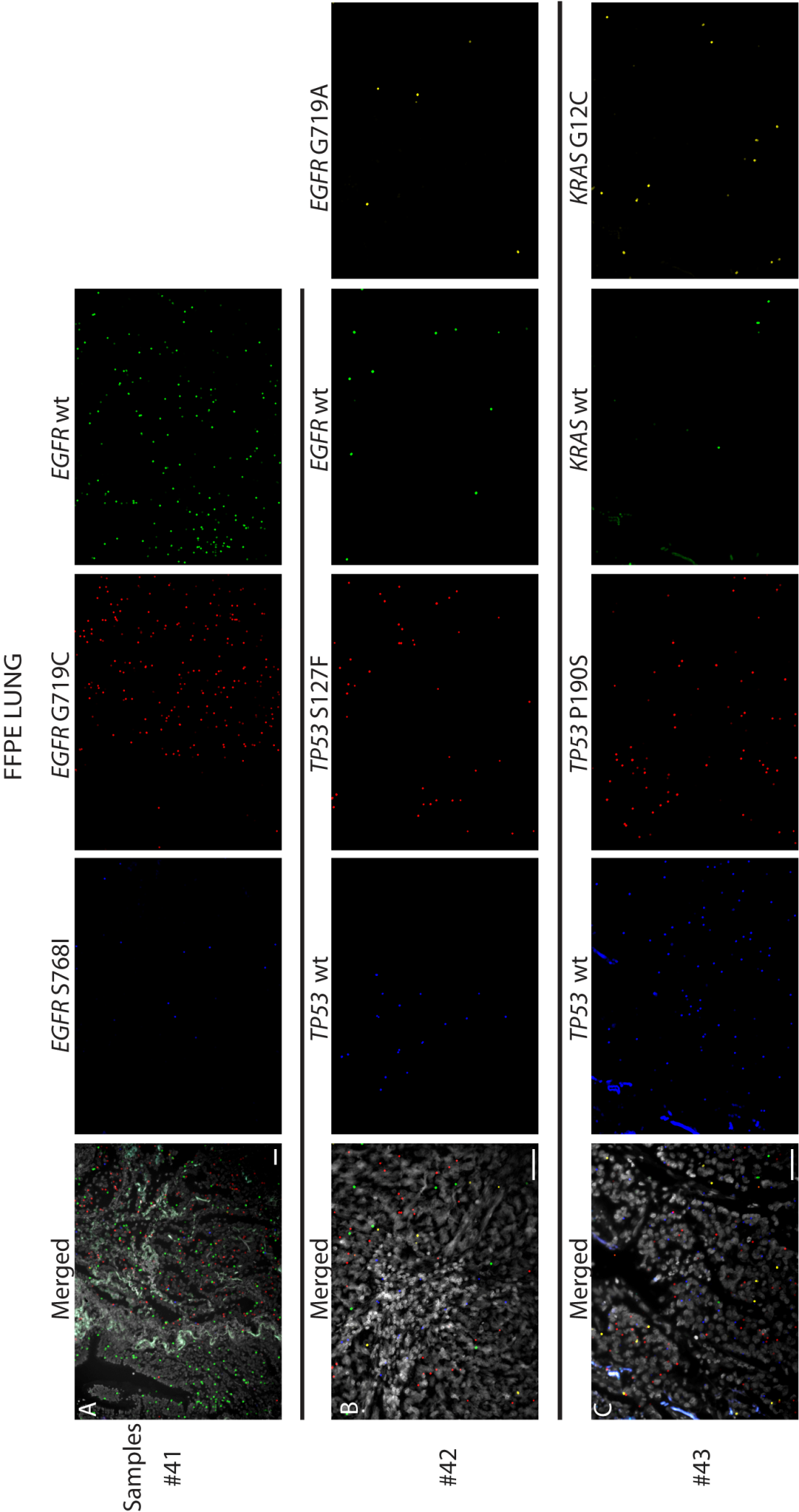
**

**Supplementary Figure 11: FFPE lung tumor tissues with (A) *EGFR* S768I and G719C mutations (wild-type:green, red:G719C, blue:S768I), (B) *TP53* S127F and *EGFR* G719A mutations (green RCPs represent wild-type *EGFR*, yellow RCPs mutant *EGFR* G719A, blue RCPs wild-type *TP53* and red RCPs represent mutant *TP53* S127F) and (C) *TP53* P190S and G12C *KRAS* mutations (green RCPs represent wild-type *KRAS*, yellow RCPs mutant *KRAS* G12C, blue RCPs wild-type *TP53* and red RCPs represent mutant *TP53* P190S).** The numbers (#41-43) represent the case numbers in **Table 1**. The images are presented in a merged format as well as in respective color to show the distribution of the target transcripts. Nuclei are shown in grey. Scale bar, 5­­­­0 µm.

**Supplementary Figure 12**


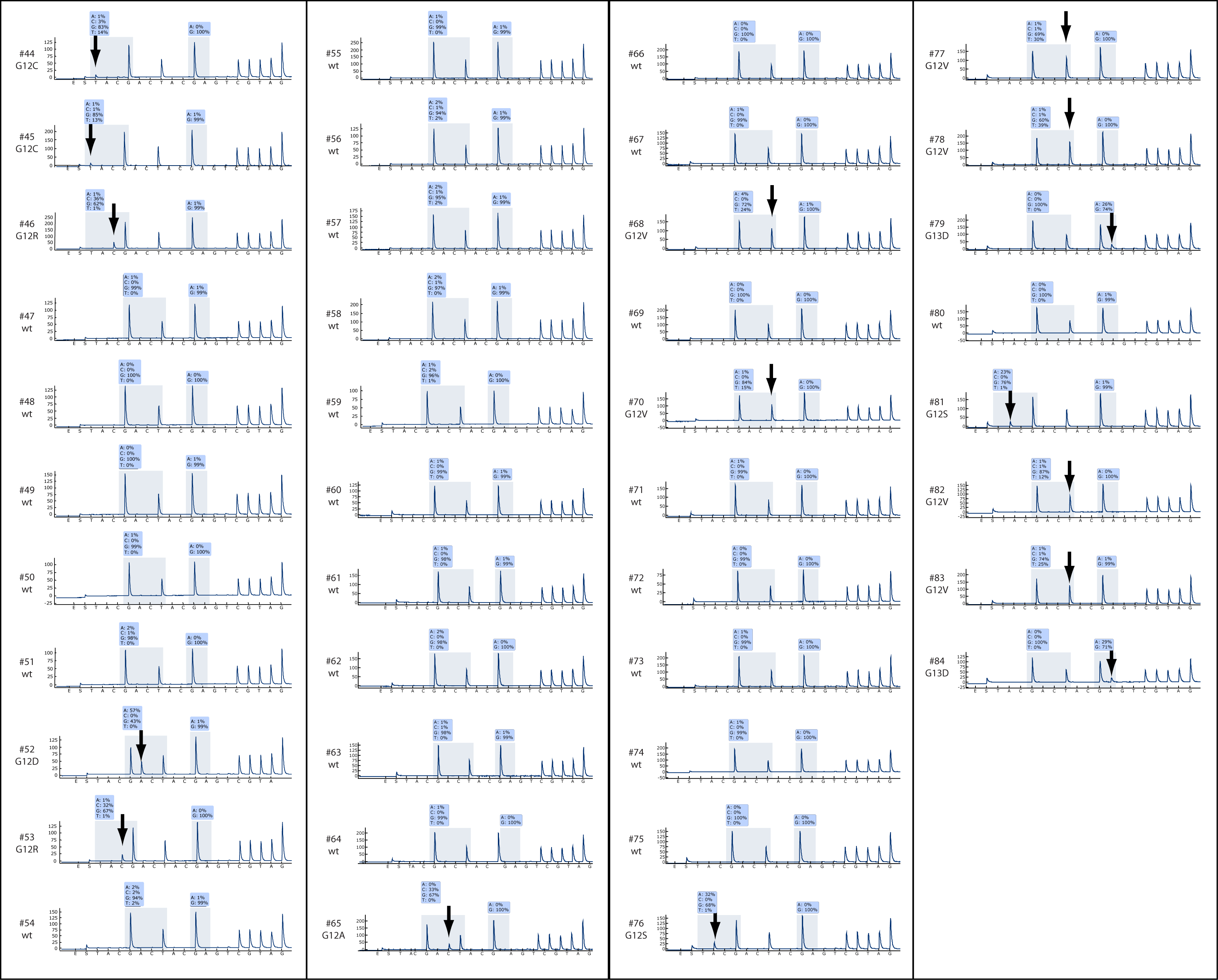


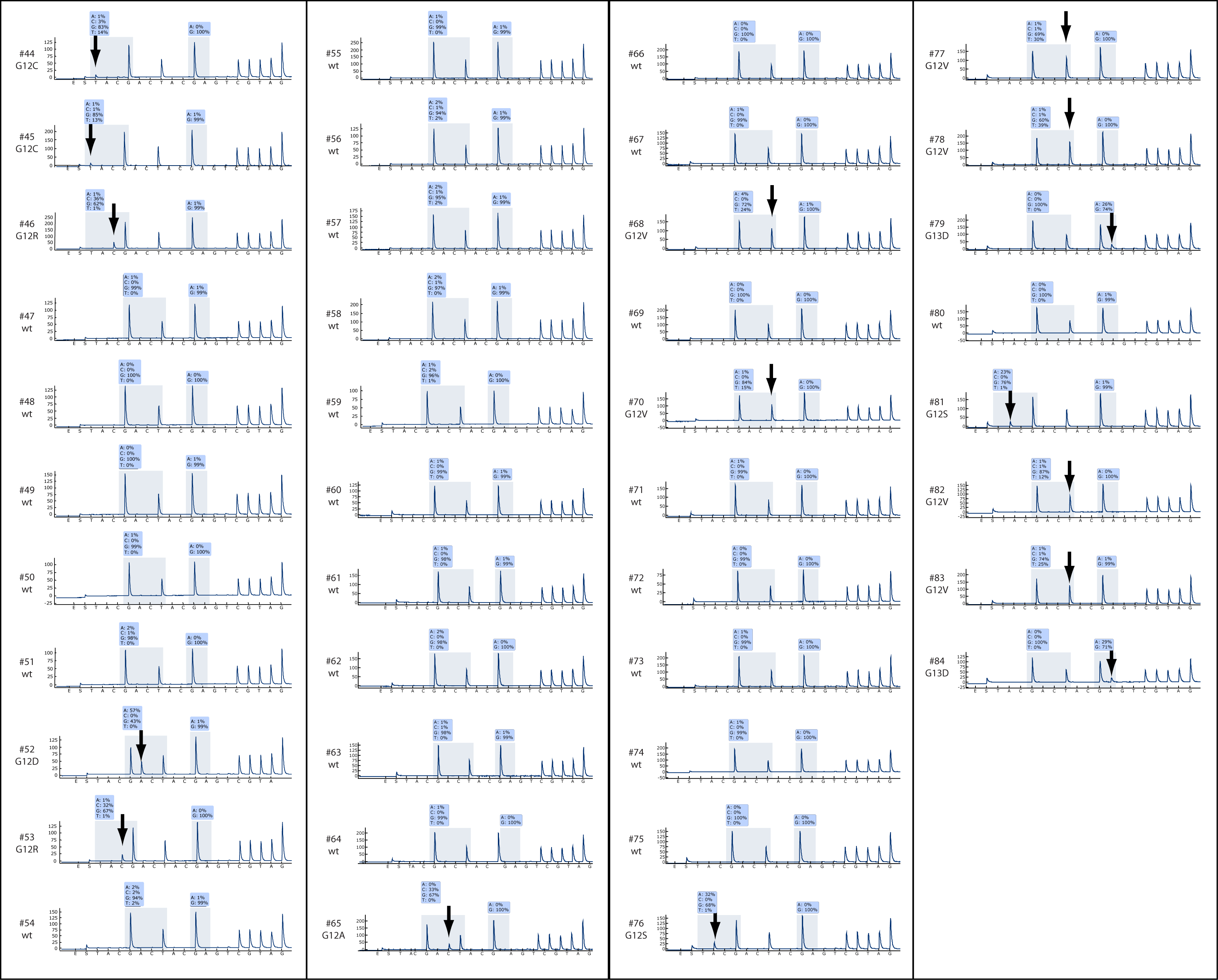


**Supplementary Figure 12: *KRAS* codon 12 and 13 were analyzed by pyrosequencing for validation of the *in situ* genotyping results.** The numbers (#44-84) represent the case numbers in **Table 2** and correspond to lung FFPE cancer tissues (#44-51), colon imprint samples (#52-59) and FFPE tissue samples from a colon TMA (#60-84). Mutations are indicated with black arrows.

**Supplementary Figure 13**


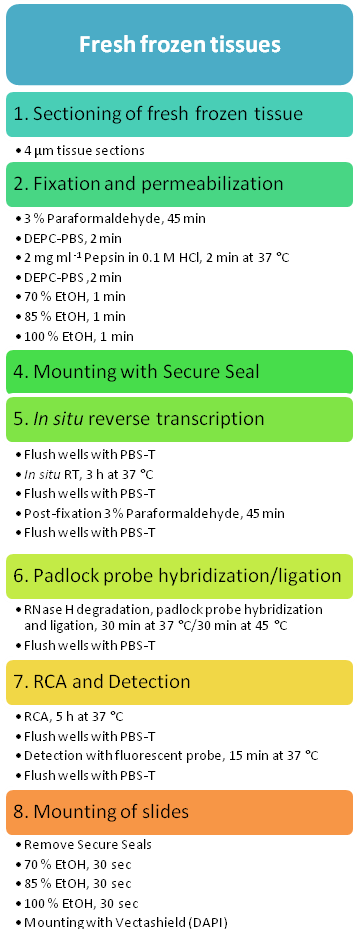

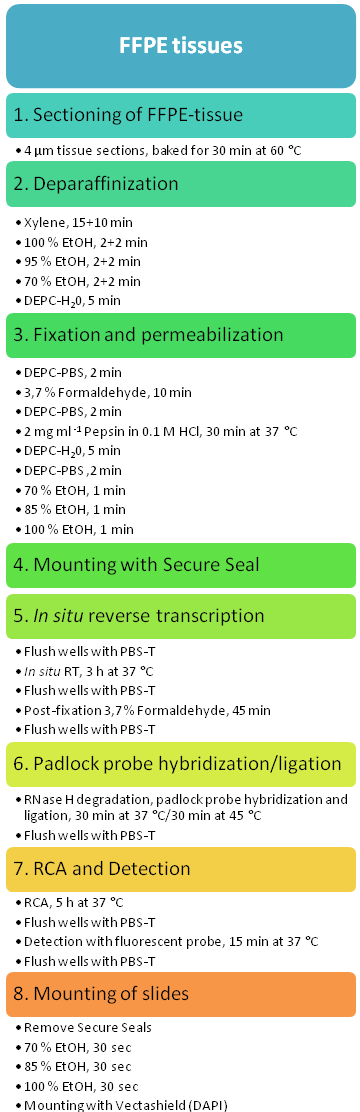

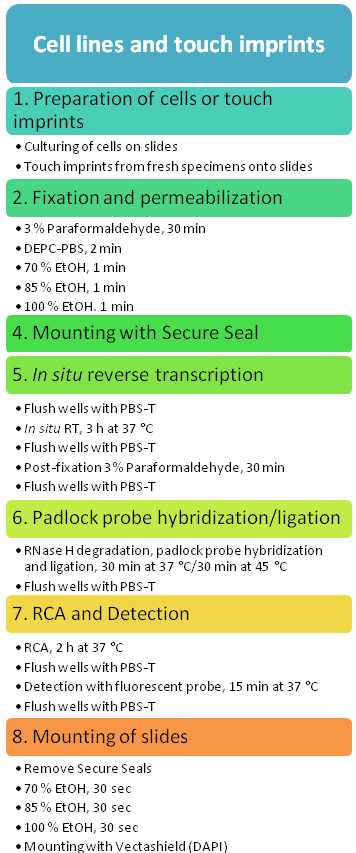


**Supplementary Figure 13: Flowcharts describing the experimental protocols that were applied for *in situ* mutation detection with padlock probes and RCA for cell lines, touch imprints, fresh frozen tissues and FFPE tissues.**

**Supplementary Table 1: Oligonucleotide sequences**

| **Primers** |  | **Sequences (5´ - 3´)** |
| --- | --- | --- |
| P-KRAS-c12/13^b^ |  | T+GT+AT+CG+TC+AA+GG+CACTCTT |
| P-KRAS-c12/13-II^a^ |  | C+CT+CT+AT+TG+TT+GG+ATCATATTCGTC |
| P-KRAS-Q61H^b^ |  | A+TG+TA+CT+GG+TC+CC+TCATTGC |
| P-EGFR-L858R^b^ |  | T+CT+TT+CT+CT+TC+CG+CACCCAG |
| P-EGFR-S768I^b^ |  | G+GC+GG+CA+CA+CGTGGGGGTTG |
| P-EGFR-G719C/A^b^ |  | C+CT+TA+TA+CA+CC+GT+GCCGAAC |
| P-TP53-S127F^b^ |  | A+GT+TG+GC+AA+AA+CA+TCTTGTTGAGGG |
| P-TP53-P190S^b^ |  | T+TC+CT+TC+CA+CT+CG+GATAAGATGCTG |
| P-ACTB^b^ |  | G+TG+GA+CG+GG+CG+GC+GGATCGGCAAAG |
| P-ACTB-II^b^ |  | A+TC+AT+CC+AT+GG+TG+AGCTGGCGGCGG |
| **Padlock probes** |  | **Sequences (5´ - 3´)** |
| PP-KRAS-wt1^a^ *(DP-1)* |  | GTGGCGTAGGCAAGATCCTAGTAATC*AGTAGCCGTGACTATCGACT*GGTTCAAAGTGGTAGTTGGAGCTG |
| PP-KRAS-G12S^a^ *(DP-2)* |  | GTGGCGTAGGCAAGATTCTAGATC*CCTCAATGCACATGTTTGGCTCC*GGTTCAAGTGGTAGTTGGAGCTA |
| PP-KRAS-G12R^a^ *(DP-2)* |  | GTGGCGTAGGCAAGATTCTAGATC*CCTCAATGCACATGTTTGGCTCC*GGTTCAAGTGGTAGTTGGAGCTC |
| PP-KRAS-G12C^a^ *(DP-2)* |  | GTGGCGTAGGCAAGATTCTAGATC*CCTCAATGCACATGTTTGGCTCC*GGTTCAAGTGGTAGTTGGAGCTT |
| PP-KRAS-wt2^a^ *(DP-1)* |  | TGGCGTAGGCAAGAGTCCTAGTAATC*AGTAGCCGTGACTATCGACT*GGTTCAAAGGGTAGTTGGAGCTGG |
| PP-KRAS-G12D^a^ *(DP-2)* |  | TGGCGTAGGCAAGAGTTCTAGATC*CCTCAATGCACATGTTTGGCTCC*GGTTCAAGGGTAGTTGGAGCTGA |
| PP-KRAS-G12V^a^ *(DP-2)* |  | TGGCGTAGGCAAGAGTTCTAGATC*CCTCAATGCACATGTTTGGCTCC*GGTTCAAGGGTAGTTGGAGCTGT |
| PP-KRAS-G12A^a^ *(DP-2)* |  | TGGCGTAGGCAAGAGTTCTAGATC*CCTCAATGCACATGTTTGGCTCC*GGTTCAAGGGTAGTTGGAGCTGC |
| PP-KRAS-wt3^a^ *(DP-1)* |  | CGTAGGCAAGAGTGCTCCTAGTAATC*AGTAGCCGTGACTATCGACT*GGTTCAAAGAGTTGGAGCTGGTGG |
| PP-KRAS-G13D^a^ *(DP-2)* |  | CGTAGGCAAGAGTGCTTCTAGATC*CCTCAATGCACATGTTTGGCTCC*GGTTCAAGAGTTGGAGCTGGTGA |
| PP-KRAS-wt4^a^ *(DP-1)* |  | GAGGAGTACAGTGCATCCTAGTAAT*CAGTAGCCGTGACTATCGACT*GGTTCAAAGGACACAGCAGGTCAA |
| PP-KRAS-Q61H^a^ *(DP-2)* |  | GAGGAGTACAGTGCACGCTAGATC*CCTCAATGCACATGTTTGGCTCC*GGTTCAAGGACACAGCAGGTCAT |
| PP-EGFR-wt1^a^ *(DP-1)* |  | GGCCAAACTGCTGGGTCCTAGTAAT*CAGTAGCCGTGACTATCGACT*GGTTCAAAGCACAGATTTTGGGCT |
| PP-EGFR-L858R^a^ *(DP-3)* |  | GGCCAAACTGCTGGGTTCTAGATA*CCTCAATGCTGCTGCTGTACTAC*GGTTCAAGCACAGATTTTGGGCG |
| PP-EGFR-wt2^a^ *(DP-1)* |  | CGTGGACAACCCCCATCCTAGTAAT*CAGTAGCCGTGACTATCGAC*TGGTTCAAAGCTACGTGATGGCCAG |
| PP-EGFR-S768I^a^ *(DP-3)* |  | CGTGGACAACCCCCATTCTAGATA*CCTCAATGCTGCTGCTGTACTAC*GGTTCAAGCTACGTGATGGCCAT |
| PP-EGFR-wt3^a^ *(DP-1)* |  | GCTCCGGTGCGTTCGTCCTAGTAAT*CAGTAGCCGTGACTATCGACT*GGTTCAAAGAGATCAAAGTGCTGG |
| PP-EGFR-G719C^a^ *(DP-2)* |  | GCTCCGGTGCGTTCGTTCTAGATC*CCTCAATGCACATGTTTGGCTCC*GGTTCAAGAGATCAAAGTGCTGT |
| PP-EGFR-wt4^a^ *(DP-1)* |  | CTCCGGTGCGTTCGGTCCTAGTAAT*CAGTAGCCGTGACTATCGAC*TGGTTCAAAGGATCAAAGTGCTGGC |
| PP-EGFR-G719A^a^ *(DP-2)* |  | CTCCGGTGCGTTCGGTTCTAGATC*CCTCAATGCACATGTTTGGCTCC*GGTTCAATGATCAAAGTGCTGGG |
| PP-TP53-wt1^a^ *(DP-3)* |  | CCCTGCCCTCAACAATTCCTTTTACGA*CCTCAATGCTGCTGCTGTACTAC*TCTTCGACTTGCACGTACTC |
| PP-TP53-S127F^a^ *(DP-4)* |  | CCCTGCCCTCAACAACTAGTATCTG*AGTCGGAAGTACTACTCTCT*TGTGCCATAAGACTTGCACGTACTT |
| PP-TP53-wt2^a^ *(DP-3)* |  | CTCCTCAGCATCTTATTCCTTTTACGA*CCTCAATGCTGCTGCTGTACTAC*TCTTCGCGATGGTCTGGCCC |
| PP-TP53-P190S^a^ *(DP-4)* |  | CTCCTCAGCATCTTACTAGTATCTG*AGTCGGAAGTACTACTCTCT*TGTGCCATAAGCGATGGTCTGGCCT |
| PP-ACTB^a^ *(DP-3)* |  | AGCCTCGCCTTTGCCTTCCTTTTACGA*CCTCAATGCTGCTGCTGTAC*TACTCTTCGCCCCGCGAGCACAG |
| PP-ACTB-II^a^ *(DP-2)* |  | AGCCTCGCCTTTGCCTTCCTTTTACGA*CCTCAATGCACATGTTTGGCTCC*TCTTCGCCCCGCGAGCACAG |
| **Detection probes** |  | **Sequences (5´ - 3´)** |
| DP-1^d^ |  | AGTAGCCGTGACTATCGACT |
| DP-2^d^ |  | CCTCAATGCACATGTTTGGCTCC |
| DP-3^c^ |  | CCTCAATGCTGCTGCTGTACTAC |
| DP-4^a^ |  | AGTCGGAAGTACTACTCTCT |
| + = LNA-modified base, underline = target complementary sequence, italic = detection probe complementary sequence | | |
| Oligonucleotides were purchased from Integrated DNA Technologies^a^, Exiqon^b^, Biomers^c^ and Eurogentec^d^. | | |

**Supplementary Table 2: Oligonucleotides applied on tissue samples for *in situ* mutation analysis**

**Supplementary Table 3: Table of mutant padlock probes and their distribution of mutant and/or wild-type signals in the tested samples**

| **Mutant padlock probes** | **Total # of samples padlock probe applied** | **# of WT samples with only signals from WT probe** |
| --- | --- | --- |
|  |  | **# of MUT samples with signals from mutant probe** |
| PP-KRAS-G12S | 44 | 39 |
|  |  | 5 |
| PP-KRAS-G12R | 41 | 39 |
|  |  | 2 |
| PP-KRAS-G12C | 46 | 40 |
|  |  | 6 |
| PP-KRAS-G12D | 45 | 40 |
|  |  | 5 |
| PP-KRAS-G12V | 45 | 35 |
|  |  | 10 |
| PP-KRAS-G12A | 45 | 41 |
|  |  | 4 |
| PP-KRAS-G13D | 45 | 39 |
|  |  | 6 |
| PP-KRAS-Q61H | 2 | 0 |
|  |  | 2 |
| PP-EGFR-L858R | 14 | 4 |
|  |  | 10 |
| PP-EGFR-S768I | 1 | 0 |
|  |  | 1 |
| PP-EGFR-G719C | 1 | 0 |
|  |  | 1 |
| PP-EGFR-G719A | 1 | 0 |
|  |  | 1 |
| PP-TP53-S127F | 1 | 0 |
|  |  | 1 |
| PP-TP53-P190S | 1 | 0 |
|  |  | 1 |

**Supplementary Note 1**

**Sample pretreatment**

Cell lines

The cell lines ONCO-DG-1, A-427, SW-480 and HCT-15 (DSMZ) were cultured in RPMI culture medium (Sigma) without L-Glutamine (Gibco) supplemented with 10% FBS (Sigma), 2 mM L-glutamine (Sigma) and 1x Penicillin-Streptomycin (PEST, Sigma). A-549 (DSMZ) was cultured in DMEM without phenol red and L-Glutamine (Gibco) supplemented with 10% FBS, 2 mM L-Glutamine and 1× PEST. HUP-T3 (DSMZ) was cultured in MEM-Eagle culture medium (Sigma) supplemented with 10% FBS, 2 mM L-glutamine and 1× PEST. All cell lines were seeded on Collagen I 8-well CultureSlides (BD BioCoat), and allowed to attach. When the cells reached the desired confluency they were fixed in 3% (w/v) paraformaldehyde (Sigma) in DEPC-treated PBS (DEPC-PBS) for 30 min at room temperature (20–23 °C). After fixation slides were washed twice in DEPC-PBS and the plastic wells were removed from the slides. The slides were thereafter dehydrated through an ethanol series of 70%, 85% and 99.5% ethanol for 1 min each. Secure-Seals were mounted over the cells and the wells were dehydrated by a brief flush with PBS-T (DEPC-PBS with 0.05% Tween-20 (Sigma)) followed by a brief permeabilization with 0.1 M HCl (in H_2_O) for 10 min at room temperature.

Tissue samples

Fresh frozen and FFPE human tumor tissues from colorectal- and lung cancer patients were obtained from the Biobank at the Department of Pathology and Cytology, Uppsala University Hospital, in accordance with the Swedish Biobank Legislation and Ethical Review Act (Uppsala Ethical Review Board approval, reference numbers 2006/325 and 2009/224). Tape transfer sections (4 µm) (Starfrost microscope slides, Instrumedics) were prepared from fresh frozen tumor samples stored at -80 °C. The slides were fixed in 3% (w/v) paraformaldehyde in DEPC-PBS for 45 min at room temperature followed by a flush in DEPC-PBS. The slides were then permeabilized with 0.01% pepsin (Sigma) in 0.1 M HCl at 37 °C for 2 min. The digestion was stopped with DEPC-treated H_2_O (DEPC- H_2_O) for 5 min followed by a wash in DEPC-PBS for 2 min. After completed pretreatments of tissues, the slides were dehydrated through an ethanol series of 70%, 85% and 99.5% ethanol for 1 min each. One fresh frozen section from each sample was H&E-stained for histopathological confirmation of the diagnosis and presence of representative tumor areas. This slide was also used for orientation prior to mutation scoring in the fluorescence microscope.

Touch imprints, prepared on Superfrost Plus microscope slides (Menzel Gläser), were obtained from fresh surgical colorectal and lung cancer specimens. After slide preparation the slides were air-dried for 1 min and thereafter stored at -80 °C. One imprint from each case was stained with Giemsa solution and used for cytological confirmation of diagnosis and presence of representative tumor cell clusters. The slides were fixed in 3% (w/v) paraformaldehyde in DEPC-PBS for 30 min at room temperature followed by a brief wash in DEPC-PBS.

FFPE tissue sections (4 µm) were placed on Superfrost Plus microscope slides and baked for 30 min at 60 °C. The slides were then deparaffinized by immersion in xylene for 15 + 10 min and thereafter gradually rehydrated through an ethanol series (2 × 2 min in 100%, 2 × 2 min in 95%, 2 × 2 min in 70%, and finally for 5 min in DEPC-H_2_O). The slides were washed in DEPC-PBS for 2 min before fixation with 3.7% formaldehyde (Sigma) in DEPC-PBS for 10 min at room temperature which was followed by another DEPC-PBS wash for 2 min. The FFPE tissue slides were then permeabilized in 2 mg ml^-1^ Pepsin (Sigma) in 0.1 M HCl at 37 °C for 30 min. The digestion was stopped by a wash in DEPC-treated H_2_O (DEPC- H_2_O) for 5 min followed by a wash in DEPC-PBS for 2 min. After completed pretreatments of tissues, the slides were dehydrated through an ethanol series of 70%, 85% and 99.5% ethanol for 1 min each.

The *KRAS* mutation status of the tissues was determined by pyrosequencing (Pyromark Q24 KRAS, Qiagen GmbH, Hilden, Germany) as described (9). *EGFR* and *TP53* mutations were characterized by direct dideoxy (Sanger) sequencing. Protocols are available upon request.

**Supplementary Note 2**

**Tissue classification and scoring of mutations**

The *in situ* mutation scoring of tissue sections was based on fluorescence spot counting and classification of the tissues by their histological appearances in a fluorescence microscope. Scoring was done by a systematic and careful microscopic analysis of the tissues. In the RCA, the long DNA concatamers are coiled into a bundle and the high local concentration of fluorophores within this bundle will appear as discrete dot-like signals, enabling discrimination between true signal and background. This high signal-to-background ratio allows for visual examination and scoring of the tissues, as well as quantification of signals using image analysis software. The tissues were carefully scrutinized for background fluorescence, thus only the RCPs that were detected in a single fluorescence channel, corresponding to the specific wavelength for the dye, were scored as true signals. This is especially important for some tissues that comprise high autofluorescence, such as lung sections, where large areas may display fluorescence in multiple channels. The tissues were inspected at various resolutions to find informative areas in the tissue sections. Importantly, the wild-type colorectal and lung tumor tissues showed very few, if any, false-positive RCPs in the tissues which made scoring relatively straightforward.

The percentage of cells that provide a signal varies substantially between tissues, but for targets such as *KRAS*, *EGFR* or *TP53*, with fairly low expression, not all cells show signals. However, for highly expressed targets, e.g. *ACTB*, signals are seen in basically every cell. Hence, the percentage of cells that produce a signal will depend on the expression level of the transcript and the integrity of the RNA in the tissue specimen. It will be very difficult to score absence of signal as a positive result, unless it is done in relation to something else (e.g. measuring a signal ratio as further described below). However, we were able to make a correct scoring of all tissue sections, even though they clearly were of quite variable quality. The effect of signal density is that it will be more difficult to find rare cell populations using low abundant transcripts as opposed to highly expressed and poor quality tissue as opposed to high quality.

We believe a uniform user friendly algorithm could be established to score and annotate patient samples as "wild-types" or "mutants". The criteria should be established in a larger training set of samples of different types and quality. We propose a simple scoring method that would be based on the ratio of numbers of mutant signals versus the total number of signals in a tumor tissue sample. We believe that a ratio of 0.1-0.2 could be reasonable, but the exact number would need to be established in a larger clinical study. I.e. a tumor tissue containing 100 mutant and 100 wild-type signals would have a ratio of 0.5, and would therefore be scored as a mutant. However, additional to this ratio one must take into account the amount of tumor cells in the tissue as well as the number of signals detected in the sample. I.e. the tissue should contain a minimum of 100 tumor cells and 10 mutant signals, to be counted as a valid mutant sample. This would be to avoid incorrect scoring of samples with poor quality that could have a ratio of 0.5, but possibly based on the counting of only a few signals in the whole tumor. An advantage of the approach is that positive scoring of mutations essentially could be made independent of the tumor cell content, since the tumor area that is used to calculate the ratio can be limited to the area where there exist mutant signals. The counting of the signals in the tissue could be performed manually, but preferably with an image analysis software. If the *in situ* technique presented herein becomes a routine assay in clinical labs, this type of scoring strategy would most certainly be necessary and of great value for interpreting the results. However, we would like to stress that these suggested criteria represent a proposed scoring method for future diagnostic purposes and was not applied in the scientific work presented herein.

An advantage with the presented *in situ* single molecule analysis is that the discrete amplified dot-like signals have very similar size and intensity within and between tissue sections. Issues with different staining intensities and patterns are sometimes problematic for regular IHC and ISH when scoring and comparing tissues. With this presented mutation detection strategy the analysis circumvents vague and challenged tissue staining patterns and detects instead bright signals clearly visualized as single objects in the sections. The approach is thereby suitable for automated image analysis, using similar algorithms as used for nuclear FISH, and automated image acquisition using fluorescence scanners.

**Supplementary Note 3**

**Assay sensitivity by serial cell dilution experiment**

To, on the one hand, test the specificity and sensitivity of the probes used in the assay and how contextual information improves the assay, and, on the other hand, evaluate how formalin fixation affect these parameters, two spike-in experiments were performed where cells from the *KRAS* mutant A-549 (G12S) cell line was spiked in different ratios into a background of the *KRAS* wild-type ONCO-DG-1 cells. In the first experiment these two cell lines were cultured on microscope slides and then fixed before analysis (mimicking fresh tissue or cytology preparations). In the second experiment, cells were mixed and pelleted, and then formalin fixed and paraffin embedded (mimicking FFPE tissue). Note that it is difficult to achieve exactly the intended ratio of cells in the two experiments (particularly in the experiment where the cells are cultured on slides after mixing), and we had no independent way of measuring the actual ratio of cells in the different preparations.

In the first experiment the *KRAS* wild-type cell line ONCO-DG-1 and the homozygous mutant cell line A-549 (G12S) were cultured separately in RPMI 1640 medium, GlutaMAX™ (Life Technologies, Carlsbad) supplemented with FBS 10% (Sigma-Aldrich, St. Louis). Upon confluency (30 million cells/sample), cells were counted using Cedex (Innovatis) and ratios of 0.1, 1, 10, 50 and 100% A-549 cells in ONCO-DG-1 were prepared together with 100% ONCO-DG-1. The cells were placed onto Superfrost Plus microscope slides and kept for 12 hours to attach. The cells were then washed twice in PBS and fixed in 3% paraformaldehyde for 30 min at room temperature. The slides were thereafter processed as previously described for cell lines. The G12S *KRAS* mutation was targeted with the specific padlock probe and applied together with the respective wild-type padlock probe. The RCPs were detected with Cy5- (wild-type) and FITC-labeled (mutant) detection probes (Supplementary Table 2). Automatic scanning was performed with a Zeiss Axio Imager Z2 equipped with a Hamamatsu ORCA-Flash 4.0 camera (2048 x 2048 pixel size) in order to image large number of cells. The individual channels of the original scanned images, consisting of single tiles, were analyzed with the CellProfiler software (version 11710). Image analysis was performed using a script in which briefly identifies single nuclei and the respective cytoplasm. The images from the FITC and Cy5 channels were filtered using the “EnhanceOrSuppressFeatures” module and the identified RCPs were related to the individual cells. Two types of analyses were performed: One in which the mutation ratio was calculated based on wild-type and mutant signal counts only, and one in which information about the spatial context was included. In this case the context is the cytoplasm of single cells. Here the mutation ratio was based on scoring individual cells as wild-type or mutant based on which signal is dominant in the individual cell. The average number of signals per cell was 1.9.

The graph above shows the result form analysis of five different cell cultures having 0%, 0.1%, 1%, 10%, 50% or 100% mutant A-549 cells (the rest being wild-type ONCO-DG-1 cells). The mutation ratio is plotted on the Y-axis. Mutation scoring without spatial context levels off at about 1% error rate (ligase specificity), while using the contextual analysis, the accuracy increases about tenfold, enabling detection of mutant cell content below 1%.

In the second experiment the effect of FFPE preservation was evaluated using sections of FFPE cell preparations with different ratios of wild-type and mutant cells. The *KRAS* wild-type cell line ONCO-DG-1 and the homozygous mutant cell line A-549 (G12S) were cultured as described above and ratios of 1% and 10% A-549 cells in ONCO-DG-1 were prepared together with 100% ONCO-DG-1. The cells were fixed in 10% neutral buffered formalin for 3 hours and pelleted by centrifugation at 2000xg for 10 minutes. The pellets were recovered, wrapped in silk paper, and further dehydrated and paraffinized using the standard protocols of the clinical histopathology lab, i.e. processed and embedded as all other FFPE tissue materials used in this paper. Sections (4 µm) were prepared from the FFPE cell blocks and placed onto Superfrost Plus microscope slides. The sections were thereafter processed as previously described for FFPE tissue samples except incubation in Glycine 2% in PBS after the postfixation step to reduce fixation-induced autofluorescence. The sections were probed, imaged, and analyzed as described above.

| **FFPE cell samples** | **# of scored cells** | | **% of scored cells** | |
| --- | --- | --- | --- | --- |
| **(A-549 : ONCO-DG-1)** | **ONCO-DG-1** | **A-549** | **ONCO-DG-1** | **A-549** |
| 100% ONCO-DG-1 | 11580 | 154 | 98,7% | 1.3% |
| 1:100 | 2946 | 61 | 98,0% | 2.0% |
| 1:10 | 2891 | 228 | 92,7% | 7.3% |

The table above presents a summary over the results from the CellProfiler analysis showing number of scored cells (ONCO-DG-1 and A-549) and the calculated ratios (in percentage) of the two cell lines identified in the different samples. In these FFPE sections we observed about 10 times less signals per cell compared to the experiment with fresh cells above (0.29 signals per cell). With this low count per cell, the contextual information from individual cells is low, and the mutation ratio did not improve notably by including spatial information into the analysis. Note that these sections are poor mimics of tumor tissue, since most solid tumors do not consist of diffuse randomly distributed rare cancer cells among a large number of normal cells, but the cancer cells rather cluster into well-defined compartments in the tissue. In Supplementary Note 2, we describe a scoring strategy based on scoring compartments of at least 100 cancer cells, which could not be used in this spike in experiment, since the cells are randomly distributed. Still, without contextual information, the pure wild-type culture scored 1.3% mutant, and the 1:100 dilution showed a value slightly higher than that and the 1:10, substantially higher.

**Supplementary Note 4**

**Comparison with published expression levels in relevant tissues**

To evaluate if the relative number of signals we observe reflects the expected abundance of these transcripts in relevant tissues, we extracted expression level data from the papers by Wang *et al.* (2008) and Hasmats (2012). They had performed RNA seq experiments on a range of tissues and cell lines, including one normal colon tissue and three each of normal lung and lung cancer tissue. RNA seq is considered to be very quantitative since it provides digital information about abundance of RNA sequences. In the table below, RPKM [(Reads Per Kilobase per Million mapped reads)](https://wiki.nci.nih.gov/pages/viewpage.action?pageId=71439191) values for the colon tissue and mean values (+/- standard deviation) for the lung tissues are presented. From these values one would expect to see similar numbers of *KRAS*, *EGFR*, and *TP53* signals and those should be about hundred times fewer than *ACTB*, which correlates well with what can be seen in our data. We also observe higher levels of detected *EGFR* transcripts in cancer cells compared to adjacent normal bronchial and alveolar epithelium.

| **Gene** | **Normal colon** | **Normal lung** | **Lung cancer** |
| --- | --- | --- | --- |
| *KRAS* | 3 | 14 (+/-6) | 10 (+/-3) |
| *EGFR* | 5 | 23 (+/-16) | 10 (+/-4) |
| *TP53* | 10 | 15 (+/-6) | 10 (+/-4) |
| *ACTB* | 5183 | 1065 (+/-330) | 1295 (+/-408) |

1. Wang ET, Sandberg R, Luo S, Khrebtukova I, Zhang L, Mayr C, Kingsmore SF, Schroth GP, Burge CB. Alternative isoform regulation in human tissue transcriptomes. Nature 456, 470-476 (2008).
2. Hasmats, J. (2012). Analysis of genetic variations in cancer. (Doctoral dissertation). Stockholm: KTH Royal Institute of Technology.
